# Supplementary material for: Cross‐ploidy hybridisation in Alpine woodrushes is associated with ecological additivity and scale‐dependent niche divergence
Source: New Phytol. 2026 Jul 20;251(6):3669–86. doi: 10.1111/nph.71443 (PMC13491294; doi:10.1111/nph.71443)
Supplement: Supplementary file 1 [file NPH-251-3669-s001.pdf]

New Phytologist

***New Phytologist* Supporting Information**

Article title: “Cross-ploidy hybridisation in Alpine woodrushes is associated with ecological additivity and scale-dependent niche divergence”
Authors: Valentin Heimer, Peter Schönswetter, Božo Frajman
Article acceptance date: 28 June 2026

The following Supporting Information is available for this article:

Fig. S1 NeighbourNet of the three study species derived from RADseq data
Fig. S2 Delta *K* plots of the STRUCTURE analyses
Fig. S3 Results of the STRUCTURE analysis including all species based on the balanced dataset
Fig. S4 Results of the STRUCTURE analysis including all species based on the full dataset
Fig. S5 Heatmap of Rho for *Luzula exspectata*
Fig. S6 Heatmap of Rho for *Luzula multiflora*
Fig. S7 Heatmap of Rho for *Luzula alpina*
Fig. S8 Isolation by distance (IBD)
Fig. S9 Isolation by environment (IBE)
Fig. S10 Results of the STRUCTURE analysis for *Luzula exspectata*
Fig. S11 Results of the STRUCTURE analysis for *Luzula multiflora*
Fig. S12 Results of the STRUCTURE analysis for *Luzula alpina*
Fig. S13 Inferred origins of range expansion
Fig. S14 Relationship between nucleotide diversity and distance to the expansion origin
Fig. S15 Results of coarse-grained niche analysis
Fig. S16 Results of the RDA of vegetation data
Fig. S17 Results of fine-grained niche analysis
Fig. S18 Box plots of fine-grained environmental data
Fig. S19 PCA of non-seed morphological characters
Fig. S20 Box plots of non-seed morphological characters
Fig. S21 Box plots of seed morphological characters
Fig. S22 PCA of seed morphological characters
Fig. S23 LDA of seed morphological characters

Table S1 *Luzula* accessions used in this study. This table is available as a separate spreadsheet
Table S2 Details of filtering applied to ddRADseq data
Table S3 Coarse-grained data used for environmental niche modelling and niche analysis
Table S4 Vegetation relevé data. This table is available as a separate spreadsheet
Table S5 Fine-grained ecological data. This table is available as a separate spreadsheet.
Table S6 Taxa with no Karrer ecological indicator values available.
Table S7 Morphometric data. This table is available as a separate spreadsheet.
Table S8 Results of Mantel tests for isolation-by-distance and isolation-by-environment
Table S9 Results of RDA for isolation-by-distance and isolation-by-environment
Table S10 Origins of range expansion
Table S11 Linear models of nucleotide diversity and distance to the origin of range expansion

New Phytologist

Table S12 Results of coarse-grained niche analysis
Table S13 RDA of coarse-grained environmental variables
Table S14 RDA of vegetation data
Table S15 Results of fine-grained niche analysis
Table S16 RDA of fine-grained environmental variables

Methods S1 Isolation-by-distance and isolation-by-environment
Methods S2 Environmental data acquisition
Notes S1 Analyses of vegetation data
Notes S2 Idiosyncratic patterns of refugial areas and range expansion

Fig. S1 NeighbourNet of the three study species based on Nei's distances computed from 49,884 SNPs present in at least 50% of samples. The three species and putative backcrosses (ALPxMUL) between *Luzula alpina* (ALP) and *L. multiflora* (MUL) are indicated with colours.

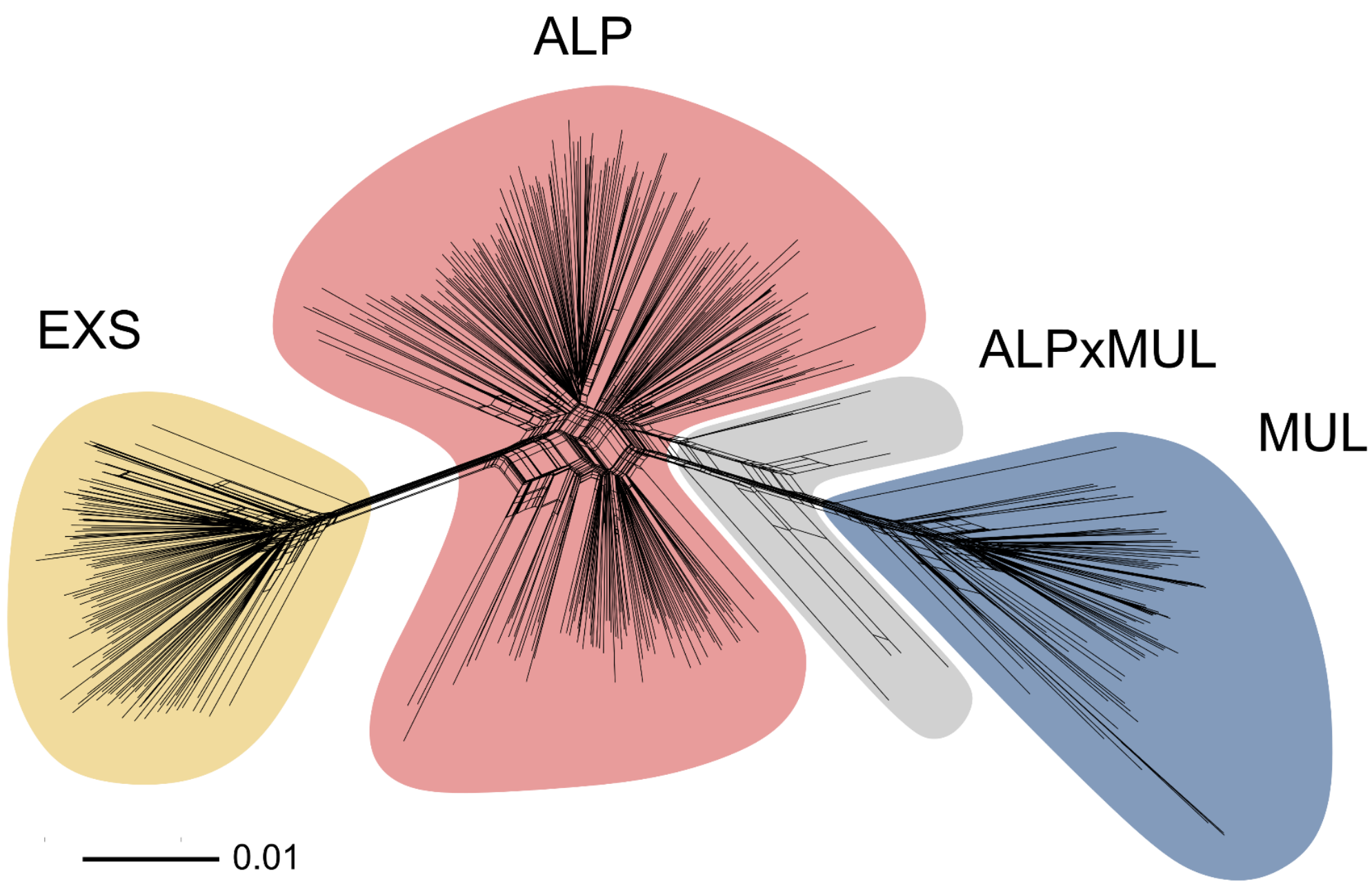

Fig. S2 Delta *K* plot based on the rate of change in the log probability between successive *K* values (numbers of genetic clusters) in the STRUCTURE analysis for the balanced dataset of 83 individuals per species (a), the full data set (b), *Luzula expectata* (EXS; c), *L. multiflora* (MUL; d) and *L. alpina* (ALP; e).

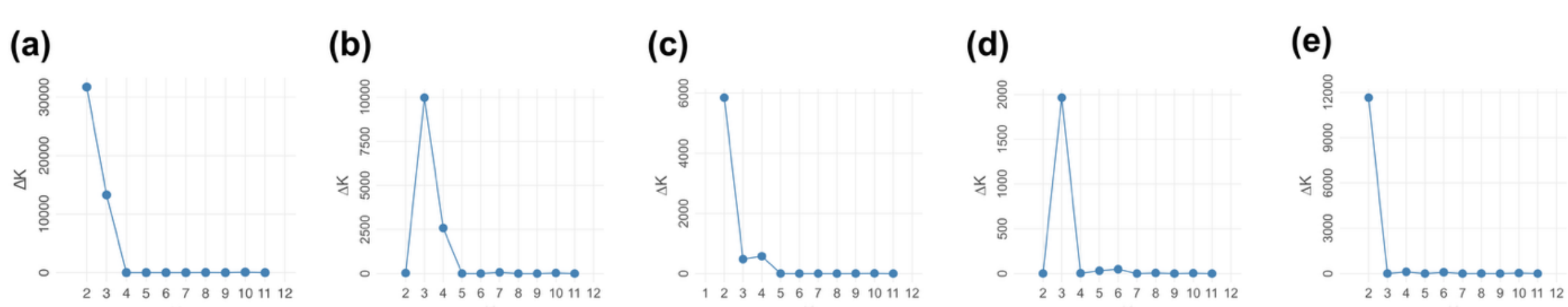

Fig. S3 STRUCTURE results for the balanced subset of 83 individuals each of *Luzula exspectata* (EXS), *L. multiflora* (MUL) and *L. alpina* (ALP) for numbers of genetic clusters *K* = 1 to 12. Species and the seven putative backcrosses between ALP and MUL (ALPxMUL) are indicated with coloured bars at the top. Numbers below bar charts are population identifiers.

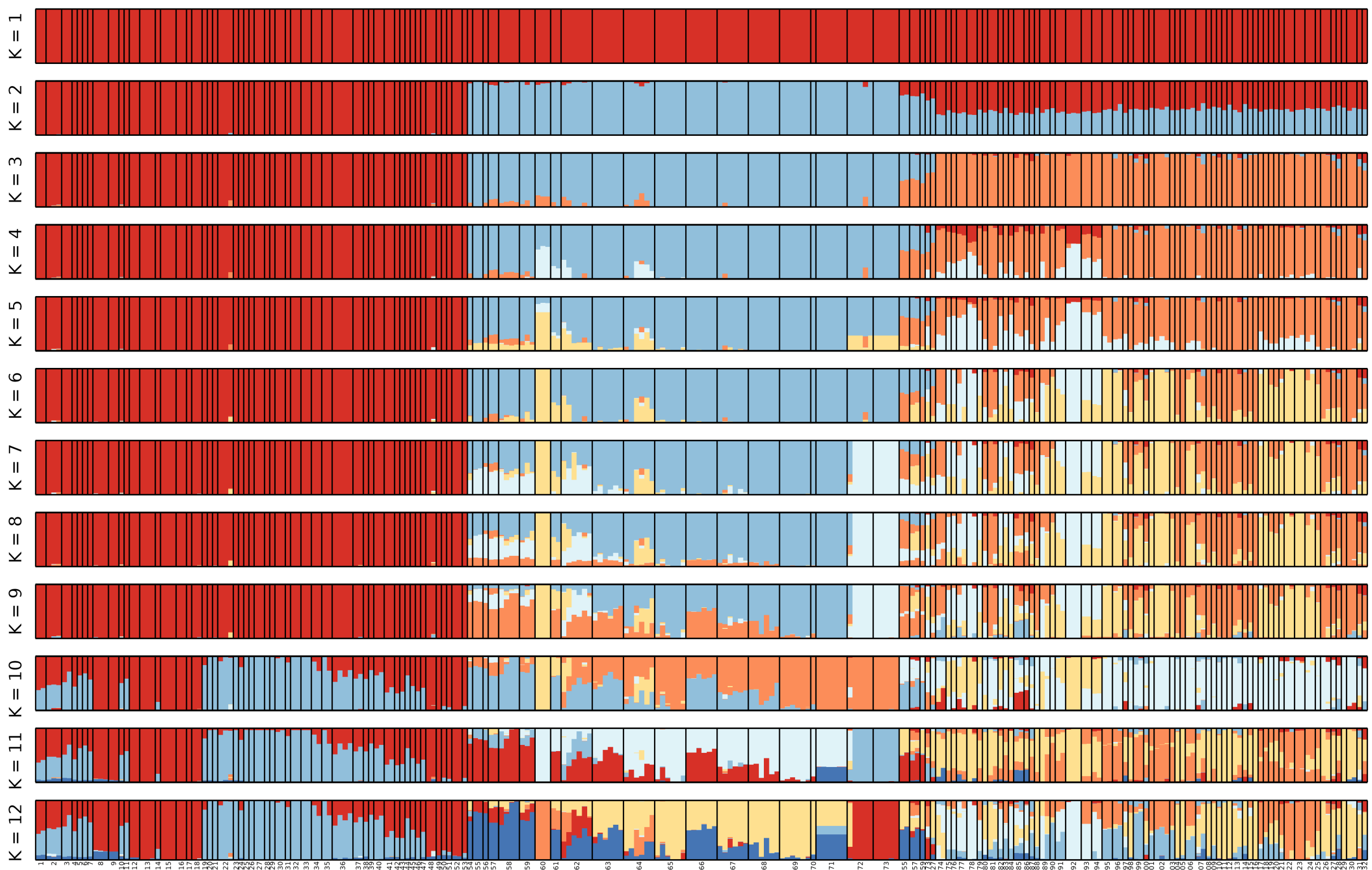

Fig. S4 STRUCTURE results for the full dataset of *Luzula exspectata* (EXS), *L. multiflora* (MUL) and *L. alpina* (ALP) for numbers of genetic clusters *K* = 1 to 12. Species and the seven putative backcrosses between ALP and MUL (ALPxMUL) are indicated with coloured bars at the top. Numbers below bar charts are population identifiers.

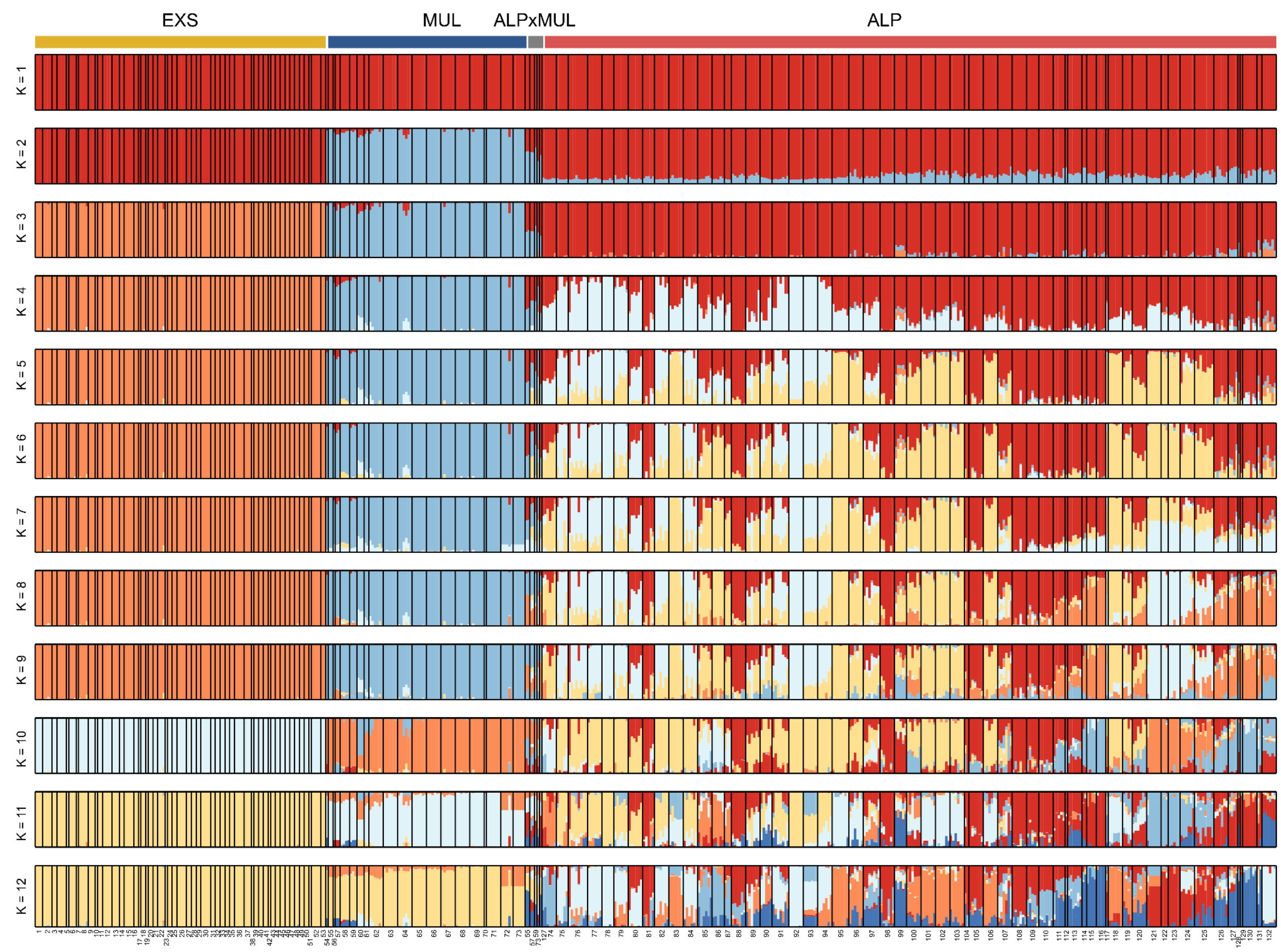

Fig. S5 Heatmap of population differentiation (Rho) for *Luzula exspectata* (EXS). Numbers on the sides of the heatmap are population identifiers and dendrograms show clustering based on Euclidean distances of Rho among populations.

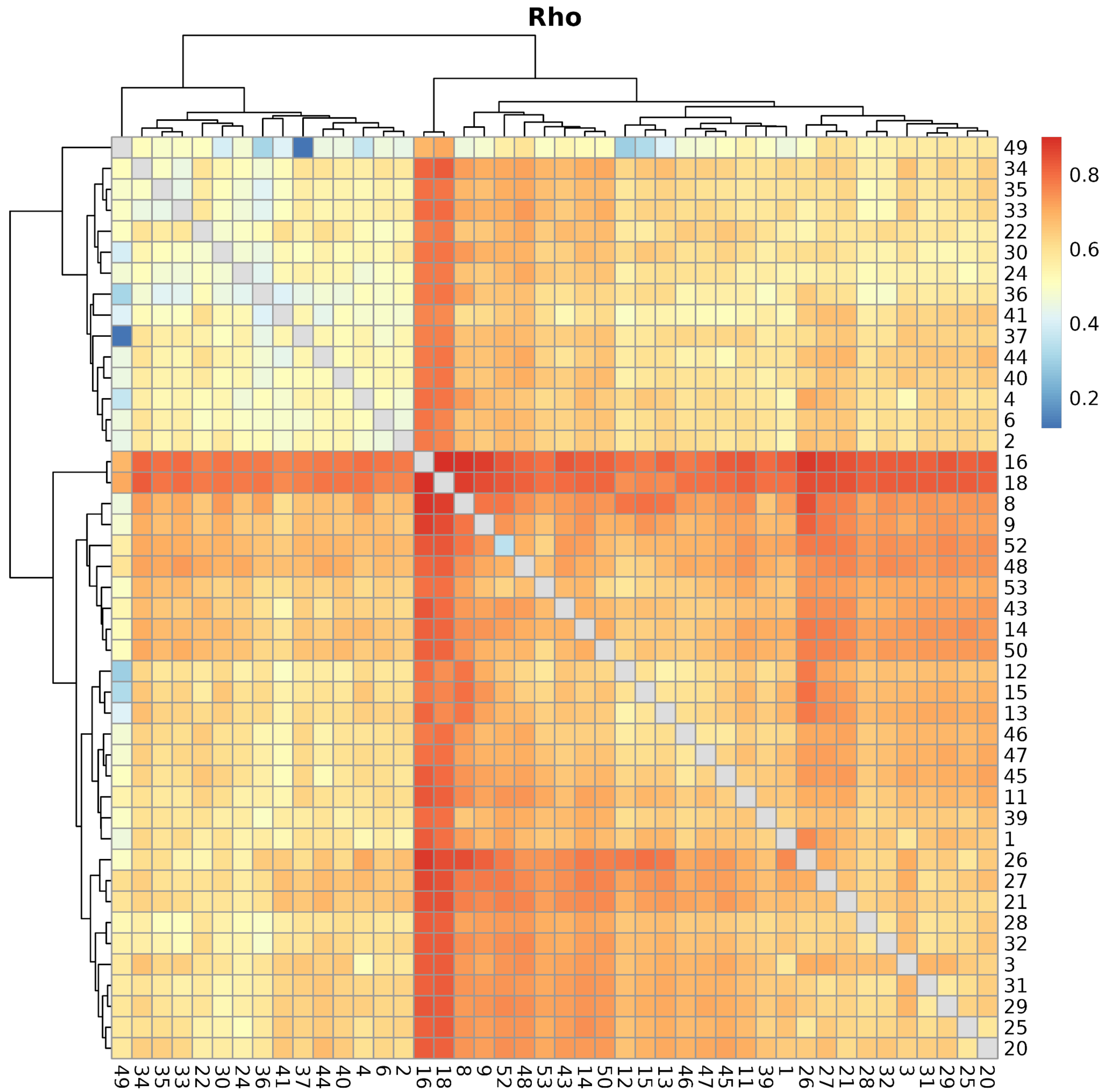

Fig. S6 Heatmap of population differentiation (Rho) for *Luzula multiflora* (MUL). Numbers on the sides of the heatmap are population identifiers and dendrograms show clustering based on Euclidean distances of Rho among populations.

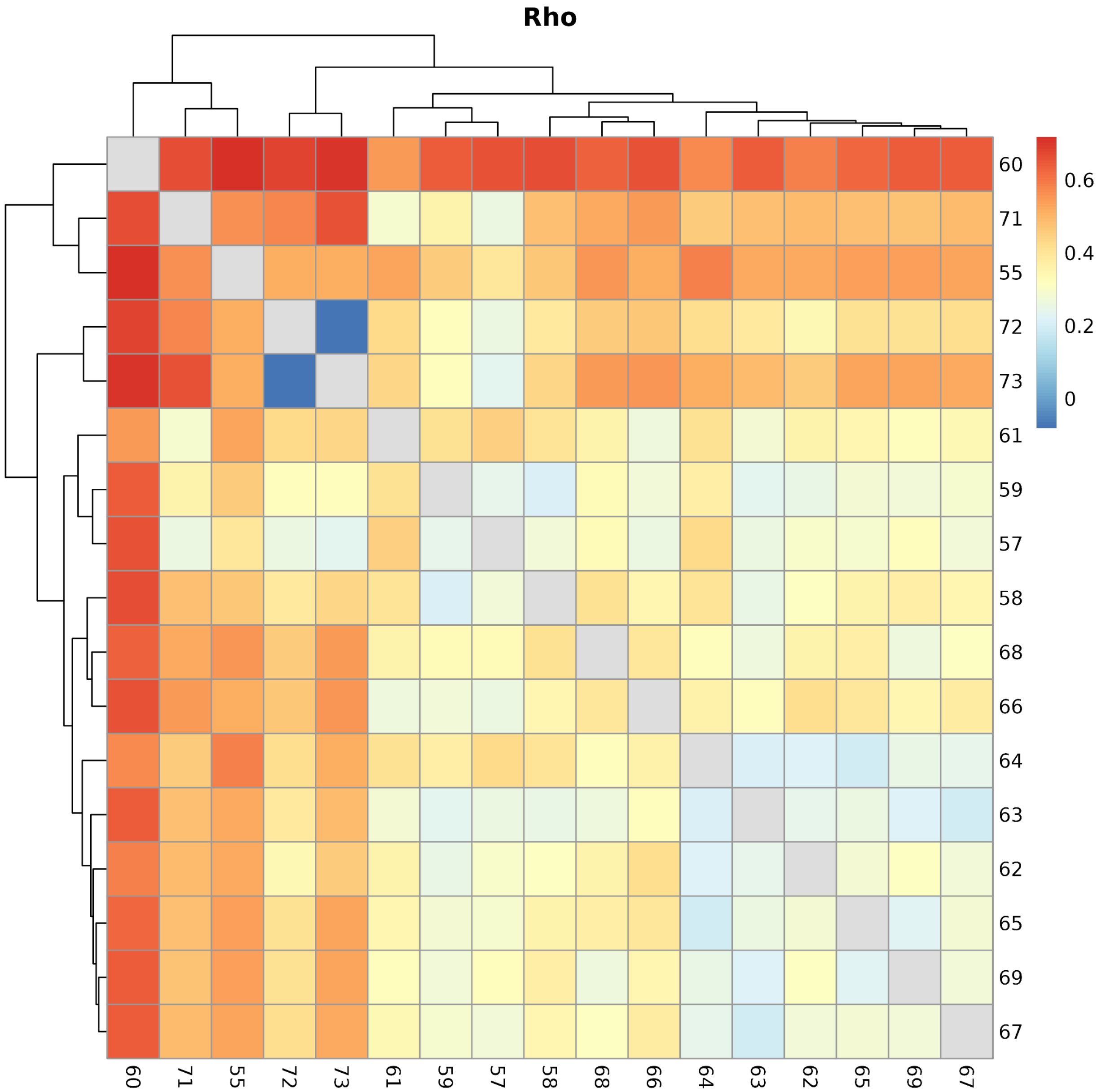

New Phytologist

Fig. S7 Heatmap of population differentiation (Rho) for *Luzula alpina* (ALP). Numbers on the sides of the heatmap are population identifiers and dendrograms show clustering based on Euclidean distances of Rho among populations.

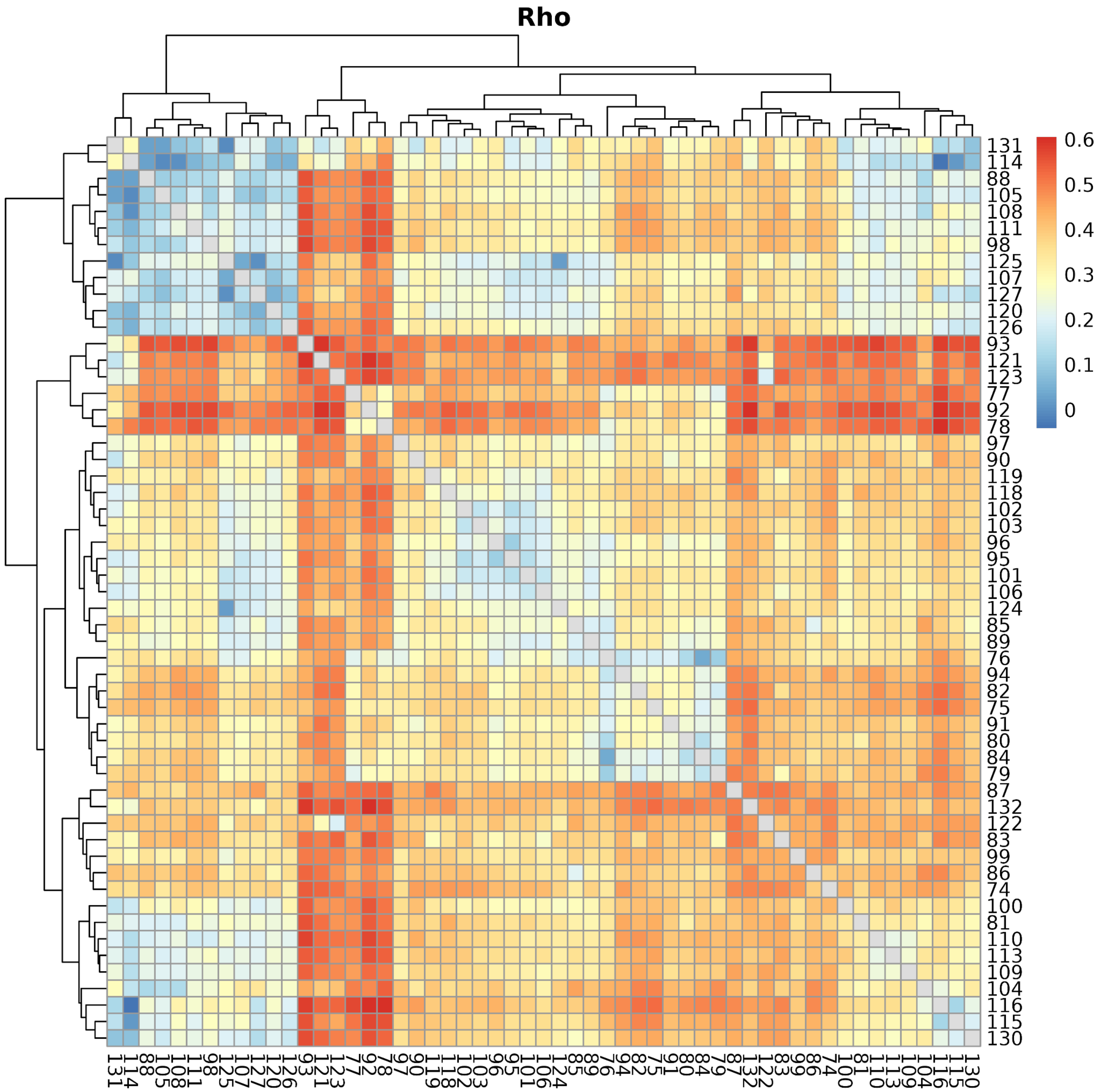

Fig. S8 Patterns of isolation by distance (IBD). IBD is shown as the relationship between genetic distance (rho/(1-rho)) to geographic distance (m) for *Luzula exspectata* (EXS; a), *L. multiflora* (MUL; b) and *L. alpina* (ALP; c). Red lines show fitted linear models and grey ribbons are 95% confidence intervals of model predictions. The test statistic (Mantel r) and *P*-value of Mantel tests with 20,000 permutations are shown in the top-left corner.

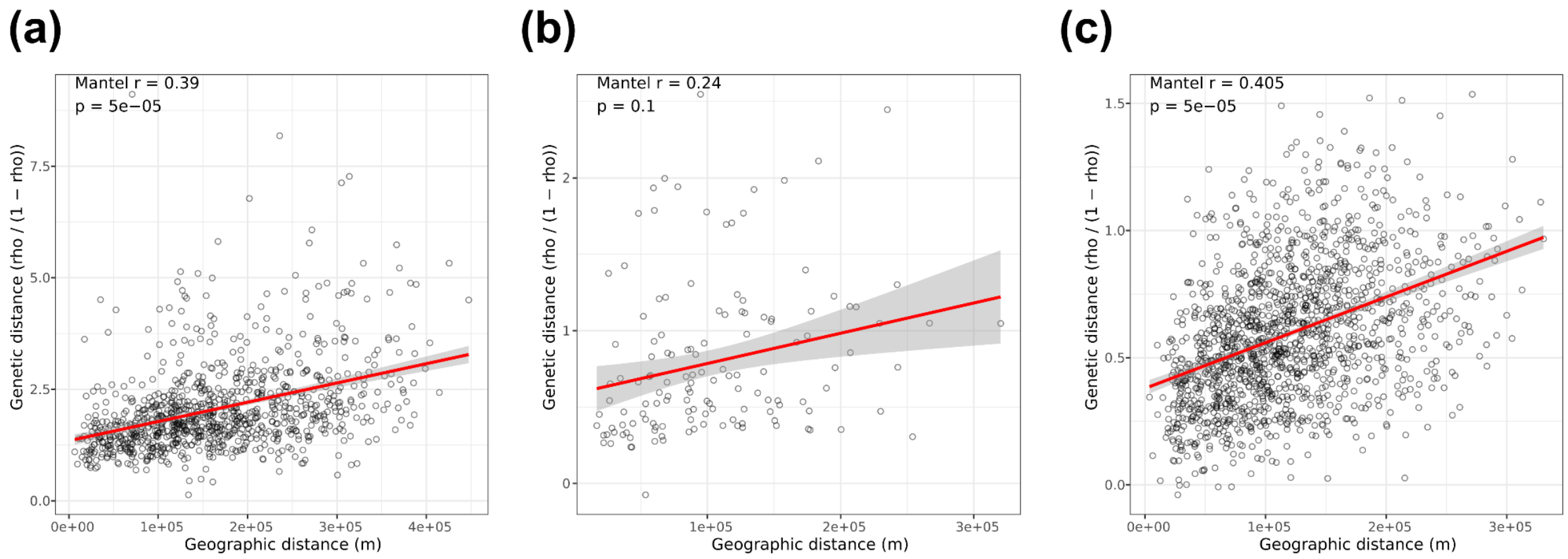

Fig. S9 Patterns of isolation by environment (IBE). IBE is shown as the relationship between genetic distance (rho/(1-rho)) to environmental distance (Euclidean distances from principal components of environmental space) for *Luzula exspectata* (EXS; a), *L. multiflora* (MUL; b) and *L. alpina* (ALP; c). Red lines show fitted linear models and grey ribbons are 95% confidence intervals of model predictions. The test statistic (Mantel r) and *P*-value of Mantel tests with 20,000 permutations while accounting for geographic structure (G ~ E | Geo) are shown in the top-left corner.

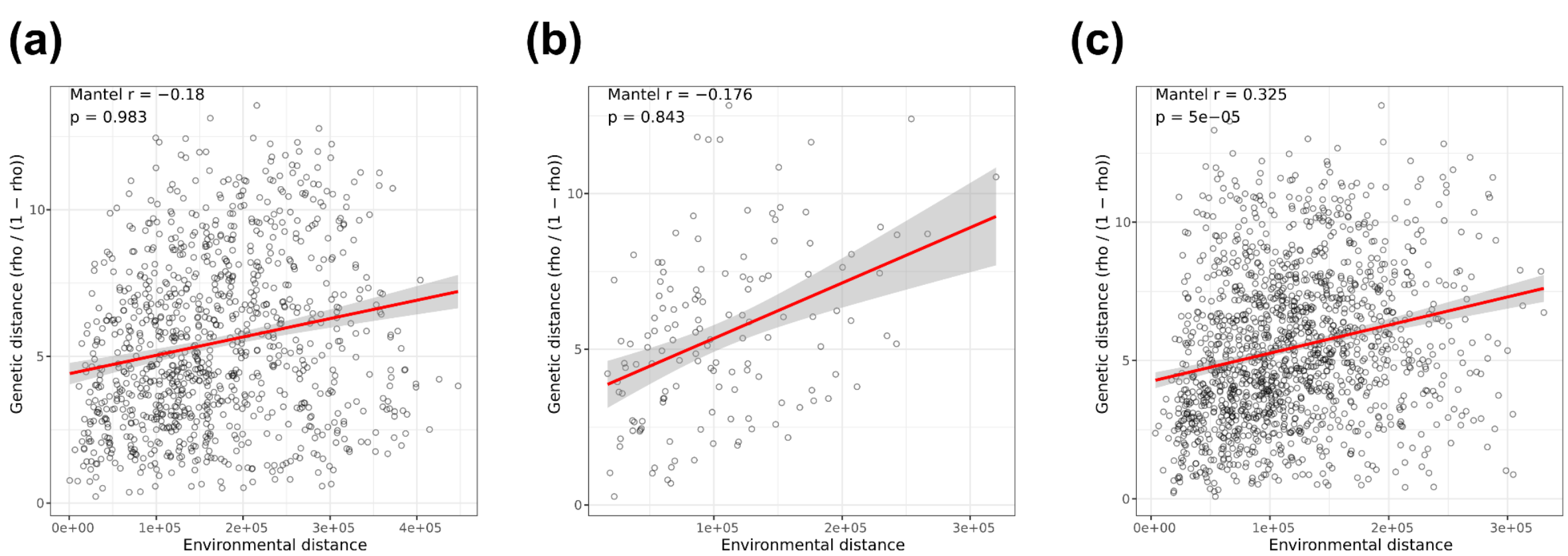

Fig. S10 STRUCTURE results for *Luzula exspectata* (EXS) for numbers of genetic clusters K = 1 to 12. Numbers below bar charts are population identifiers.

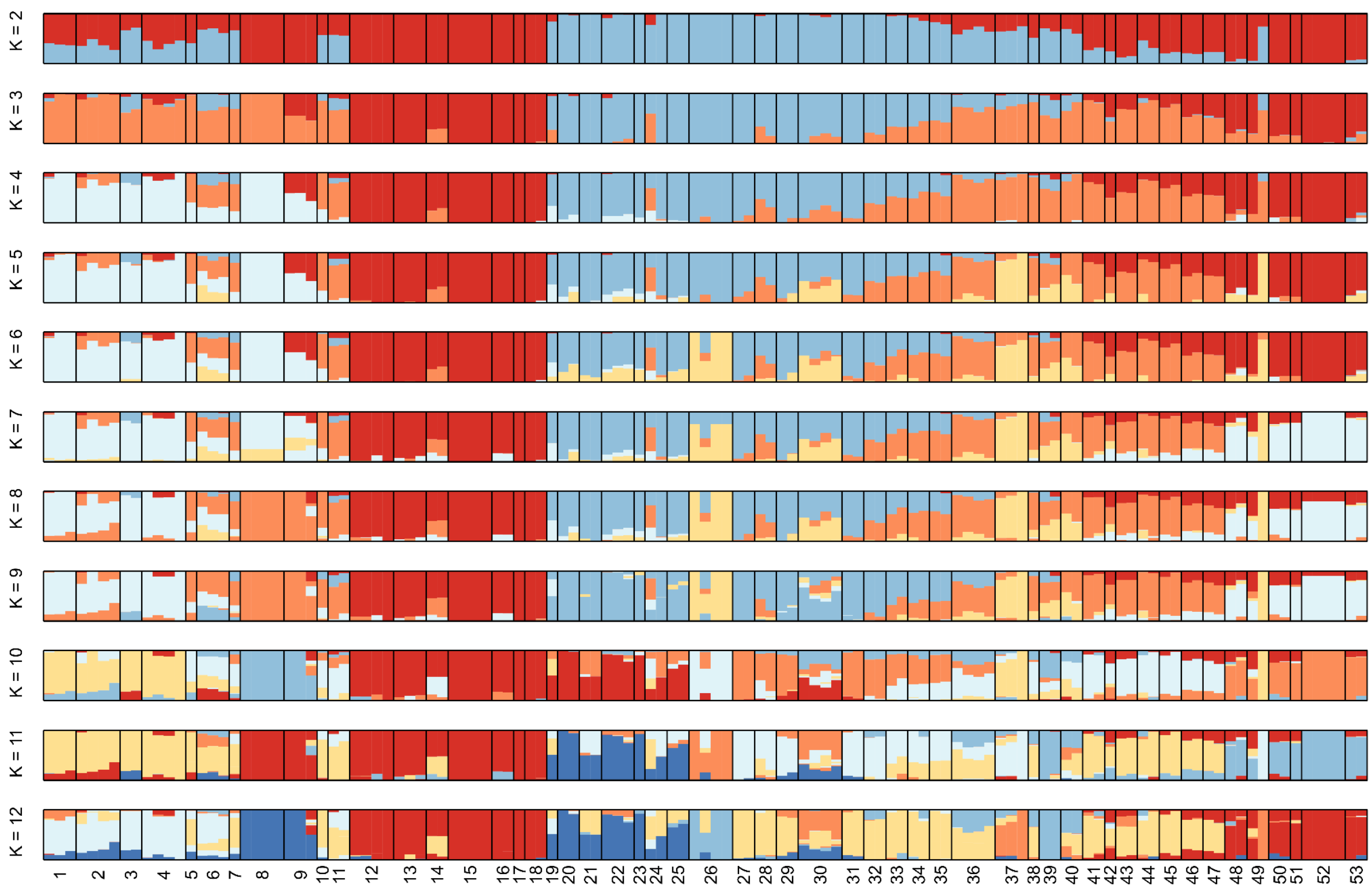

Fig. S11 STRUCTURE results for *Luzula multiflora* (MUL) for numbers of genetic clusters *K* = 1 to 12. Numbers below bar charts are population identifiers.

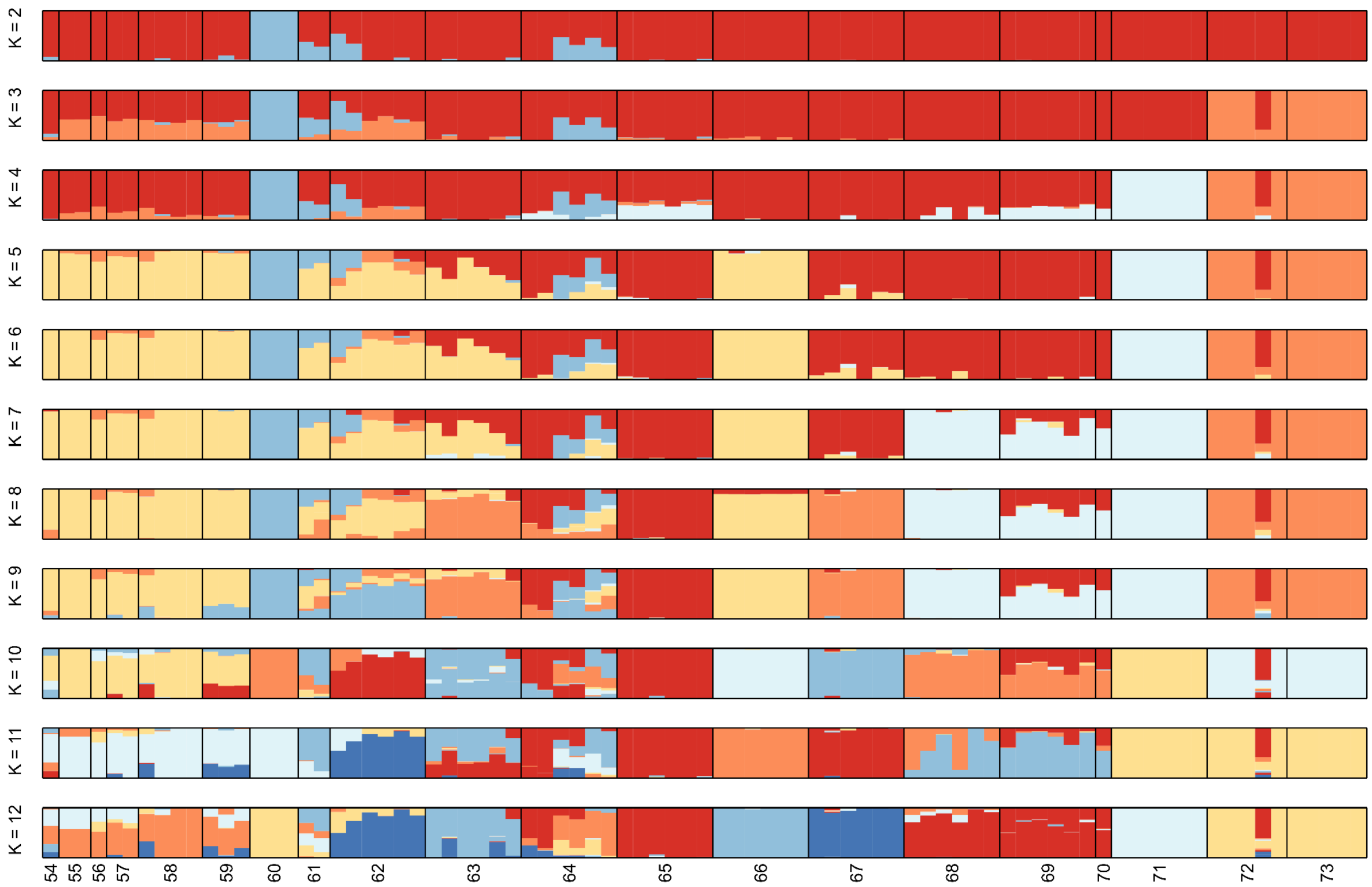

Fig. S12 STRUCTURE results for *Luzula alpina* (ALP) for numbers of genetic clusters *K* = 1 to 12. Numbers below bar charts are population identifiers.

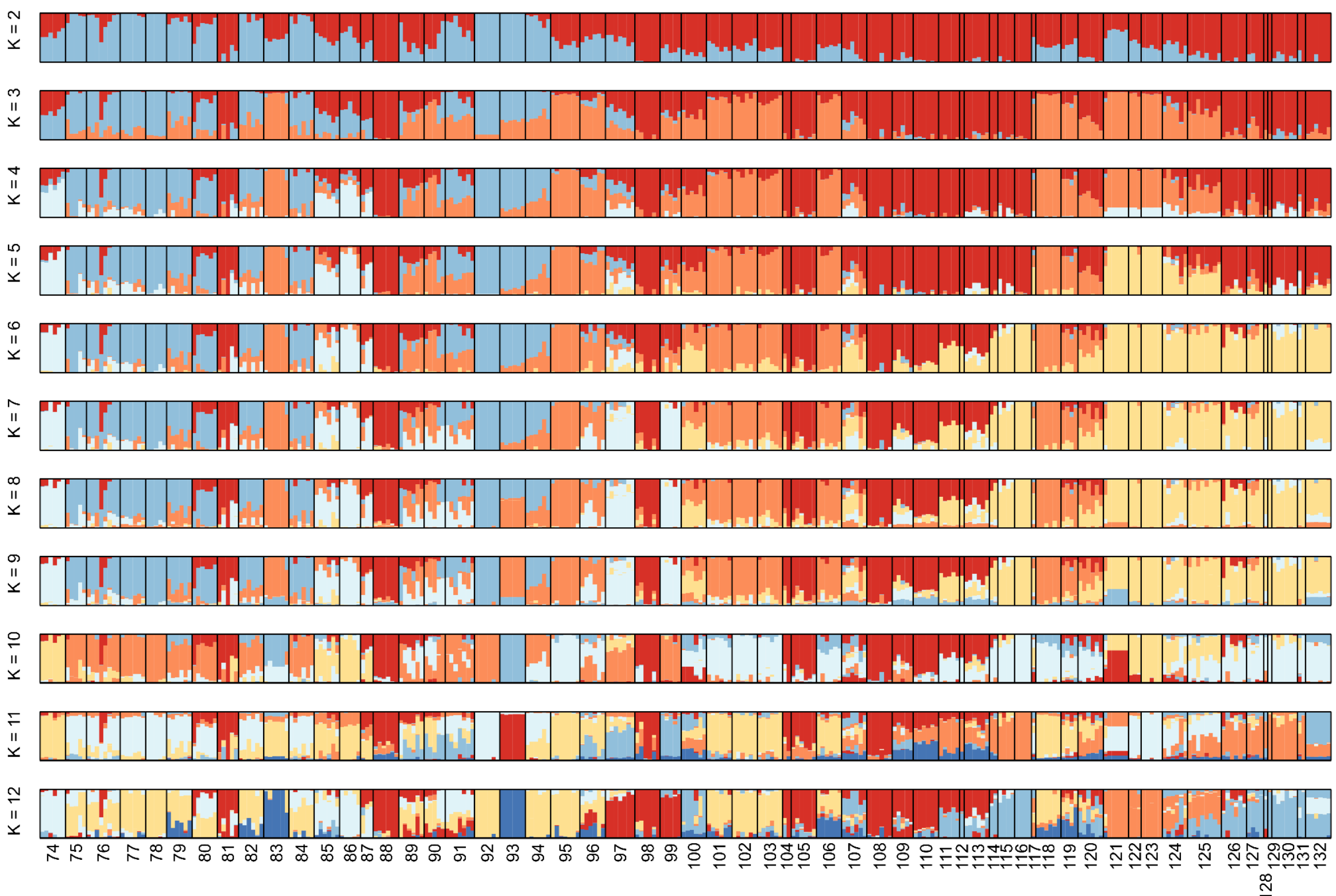

Fig. S13 Origins of range expansion. The most likely origin of range expansion inferred from the directionality index ψ is shown for either the entire species or major genetic groups defined by STRUCTURE for *Luzula exspectata* (EXS) (a), *L. multiflora* (MUL; b) and *L. alpina* (ALP) (c). Only the full species was tested for MUL as STRUCTURE clusters did not follow clear geographic patterns in this species. Brighter colour shading indicates a higher probability of an area being the origin of range expansion and the most likely origin is highlighted by an X.

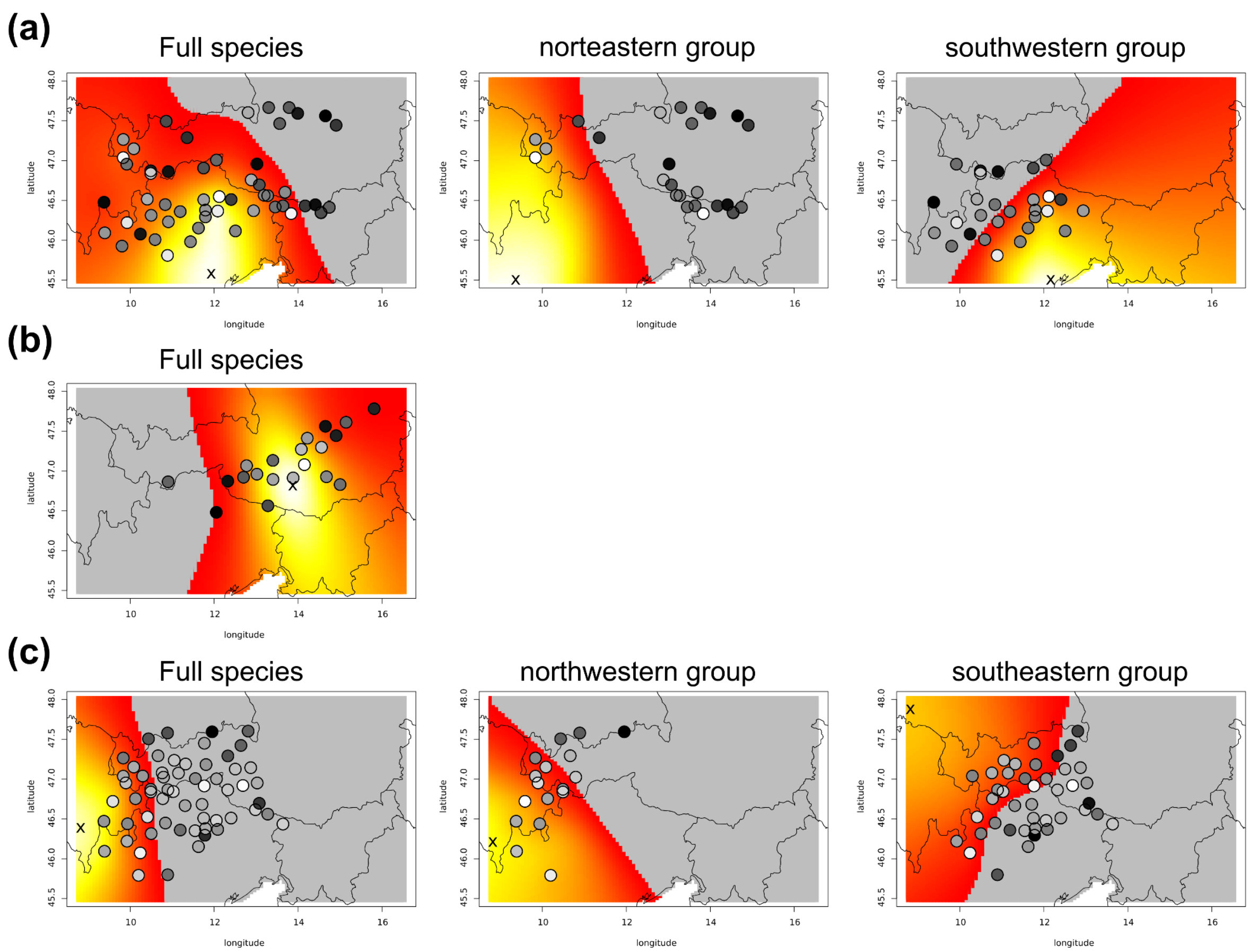

Fig. S14 Relationship between population-average nucleotide diversity (π) and geographic distance to the inferred origin of range expansion. Results are shown for *Luzula exspectata* (EXS; a), *L. multiflora* (MUL; b) and *L. alpina* (ALP; c). Blue lines show fitted linear models and grey ribbons are 95% confidence intervals of model predictions.

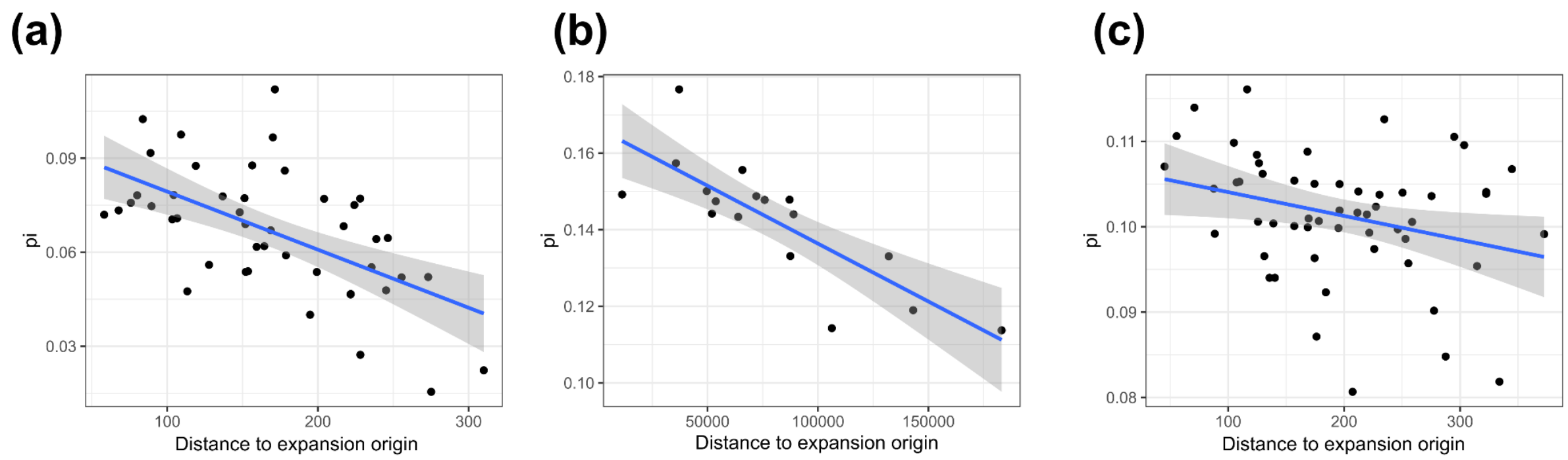

Fig. S15 Analysis of niches based on coarse-grained ecological data. (a) Pairwise comparison of ecological niches derived from climatic and edaphic ecological data in *ecospat*. Coloured regions show ecological niches for each species along the first two principal components of multivariate niche space while overlapping niches are shown in grey. The background area is shown as a red line and coloured symbols are species occurrences. (b) Variable contributions to the principal component analyses of environmental space.

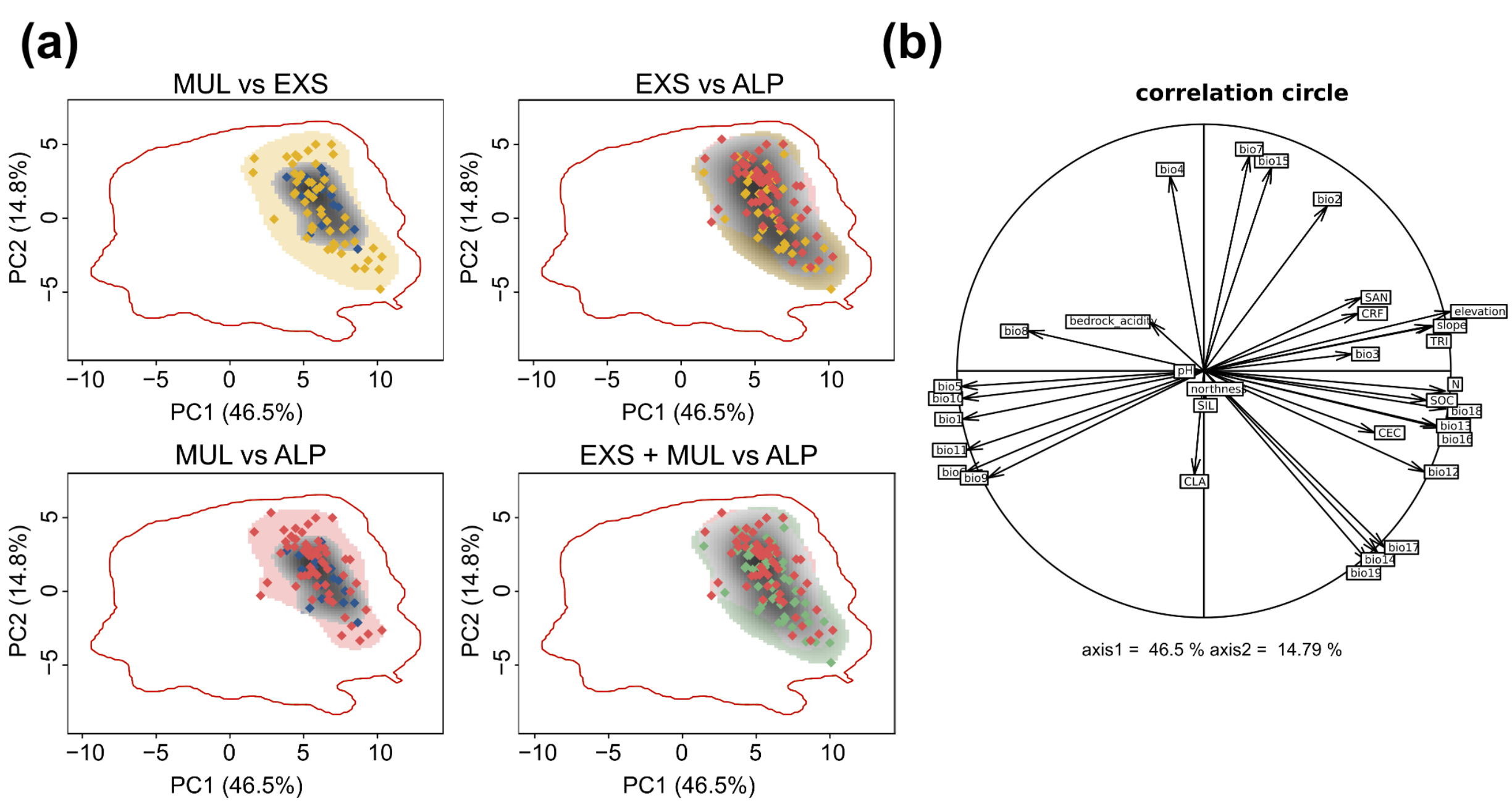

Fig. S16 Optimised redundancy analysis (RDA) of plant community composition showing the accompanying plant species most strongly differentiating between the three study species. The occurrence of each *Luzula* species shown as colored dots (*Luzula exspectata*, EXS, yellow; *L. multiflora*, MUL, blue; and *L. alpina*, ALP, red) was used as a response variable. The proportion of variation explained by each RDA axis is shown in parentheses.

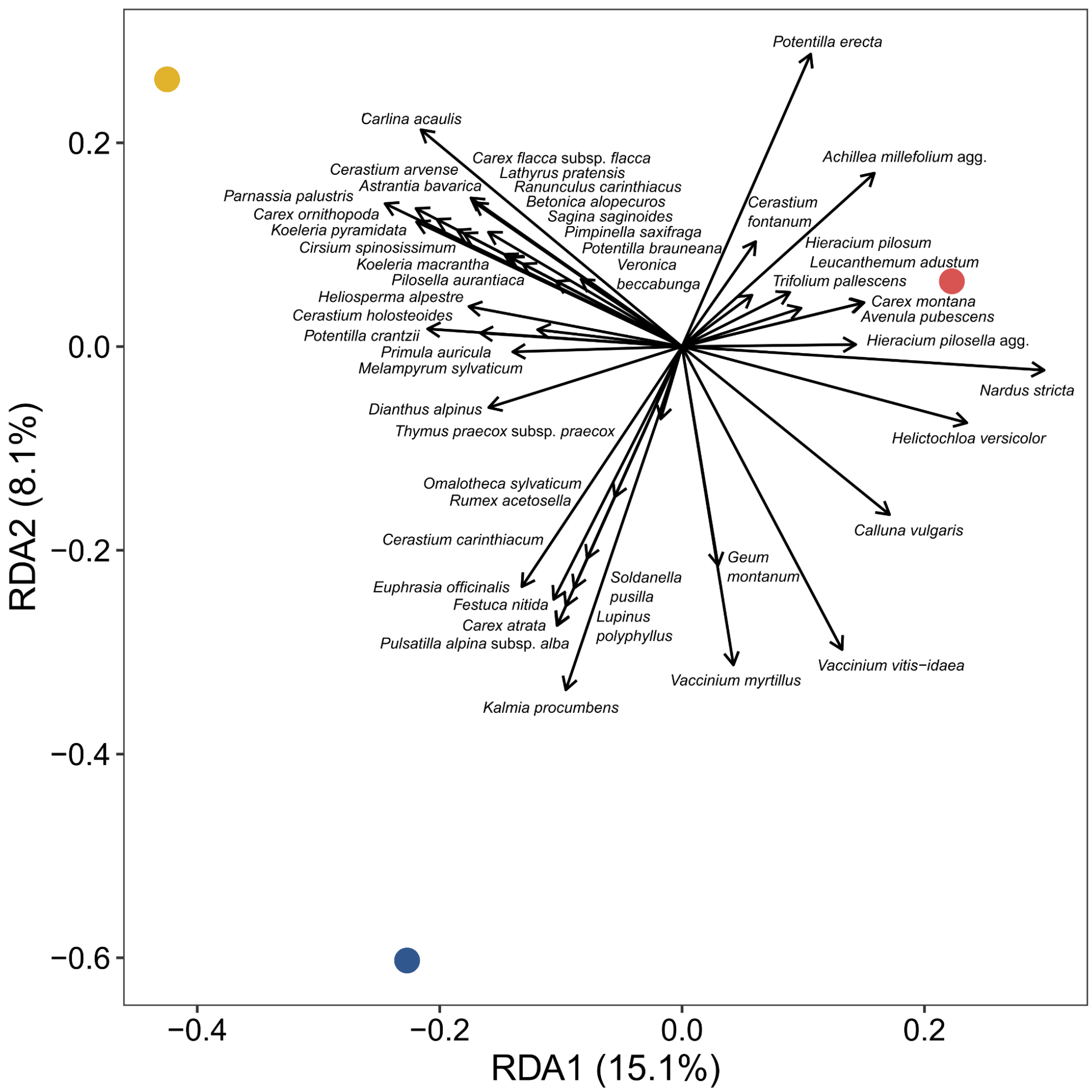

Fig. S17 Analysis of niches based on fine-grained environmental data. (a) Pairwise comparison of ecological niches computed in *ecospat*. Coloured regions show ecological niches for each species along the first two principal components of multivariate niche space while overlapping niches are shown in grey. The background area is shown as a red line and coloured symbols are species occurrences. (b) Variable contributions to the principal component analyses of environmental space.

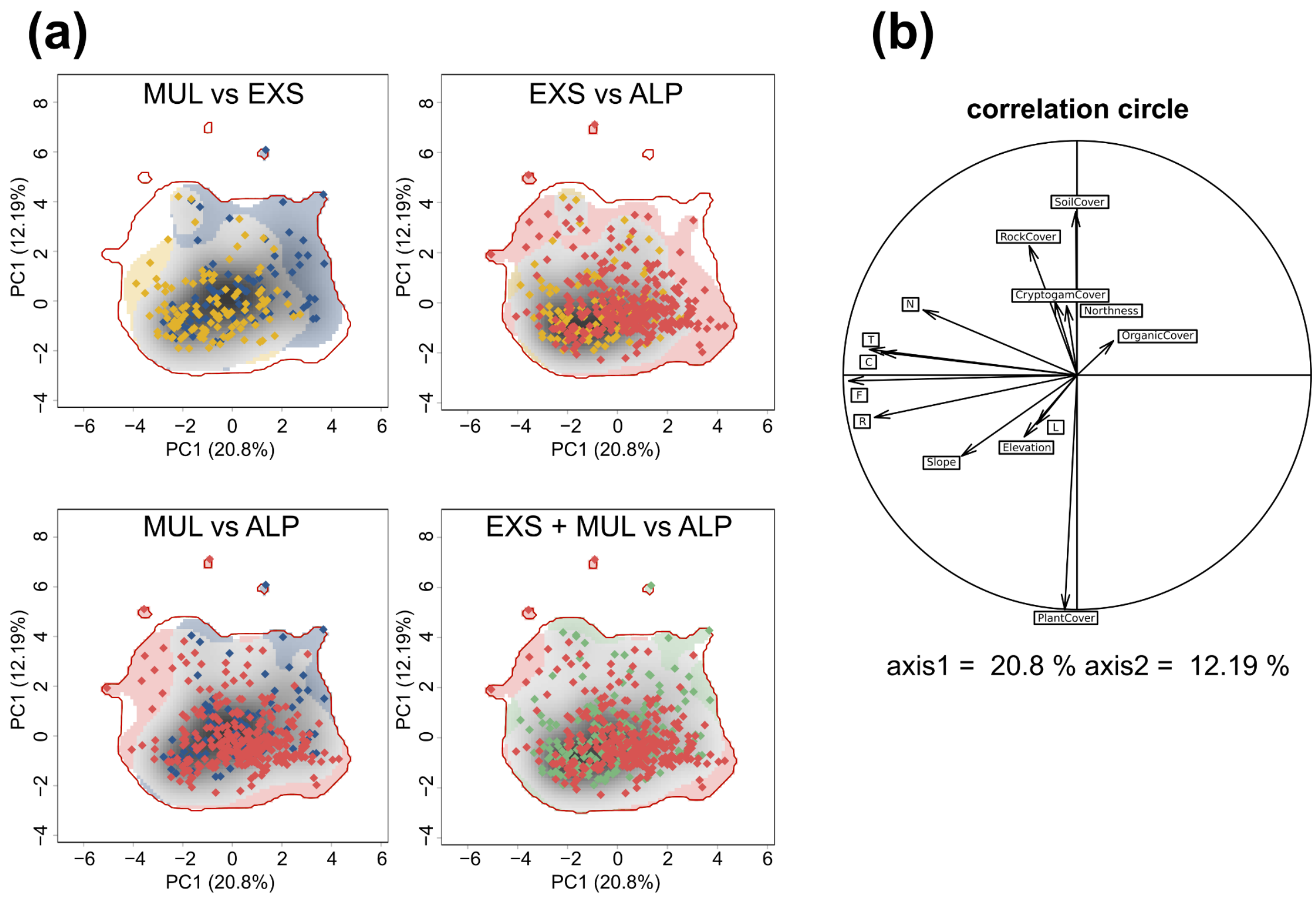

Fig. S18 Boxplots of fine-grained environmental data recorded for each individual of *Luzula exspectata* (EXS; yellow), *L. multiflora* (MUL; blue) and *L. alpina* (ALP; red). Community-weighted means of Karrer indicator values were computed from accompanying vascular plant species. Thick horizontal lines are median values, boxes are interquartile ranges (IQR) and whiskers show 1.5 times the IQR. Asterisks above brackets indicate significant mean differences according to Kruskal-Wallis test followed by Dunn's *post hoc* test (ns: not significant, * $P < 0.05$, ** $P < 0.01$, *** $P < 0.001$).

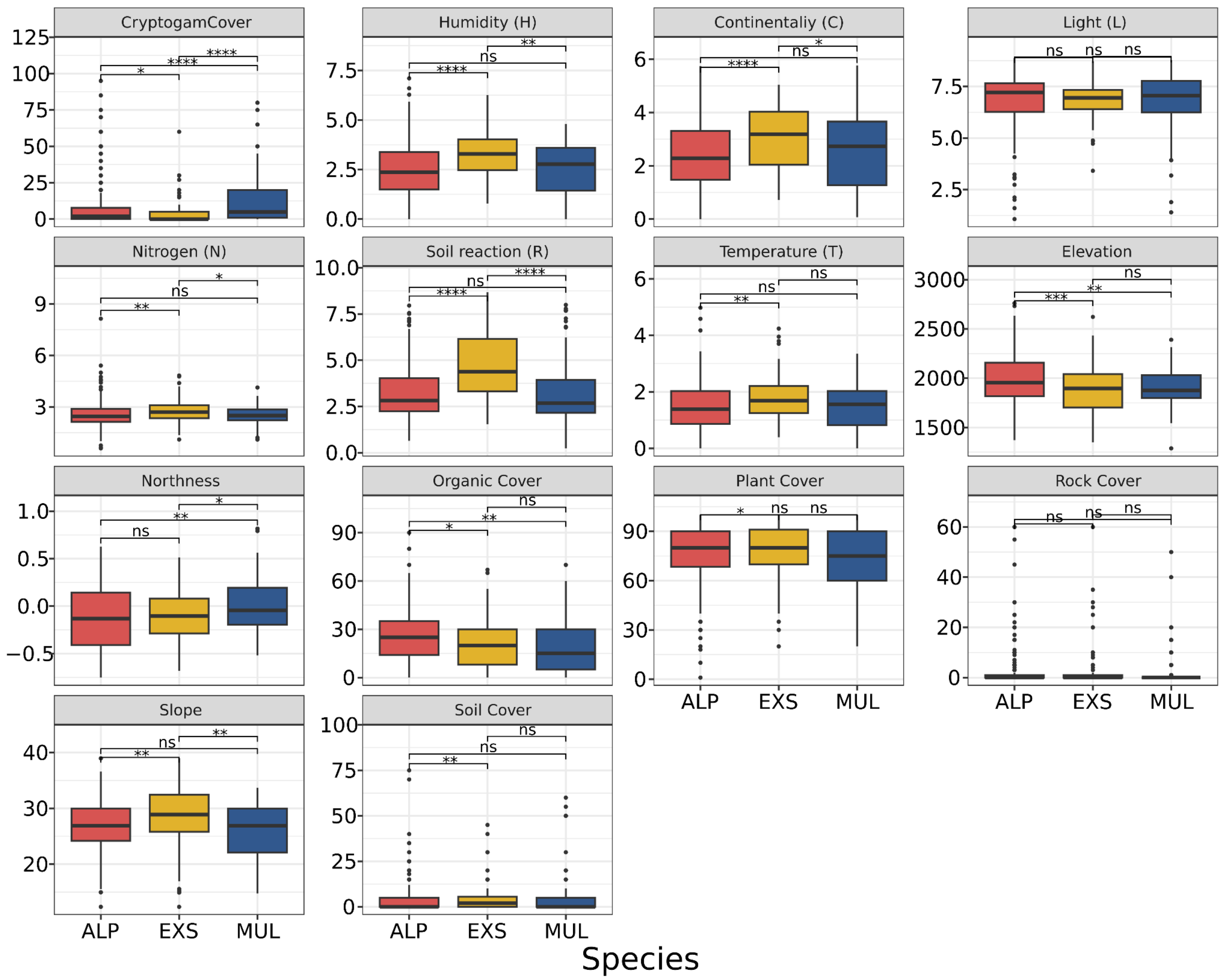

Fig. S19 Principal component analysis (PCA) of non-seed morphological characters of *Luzula exspectata* (EXS; yellow), *L. multiflora* (MUL; blue) and *L. alpina* (ALP; red).

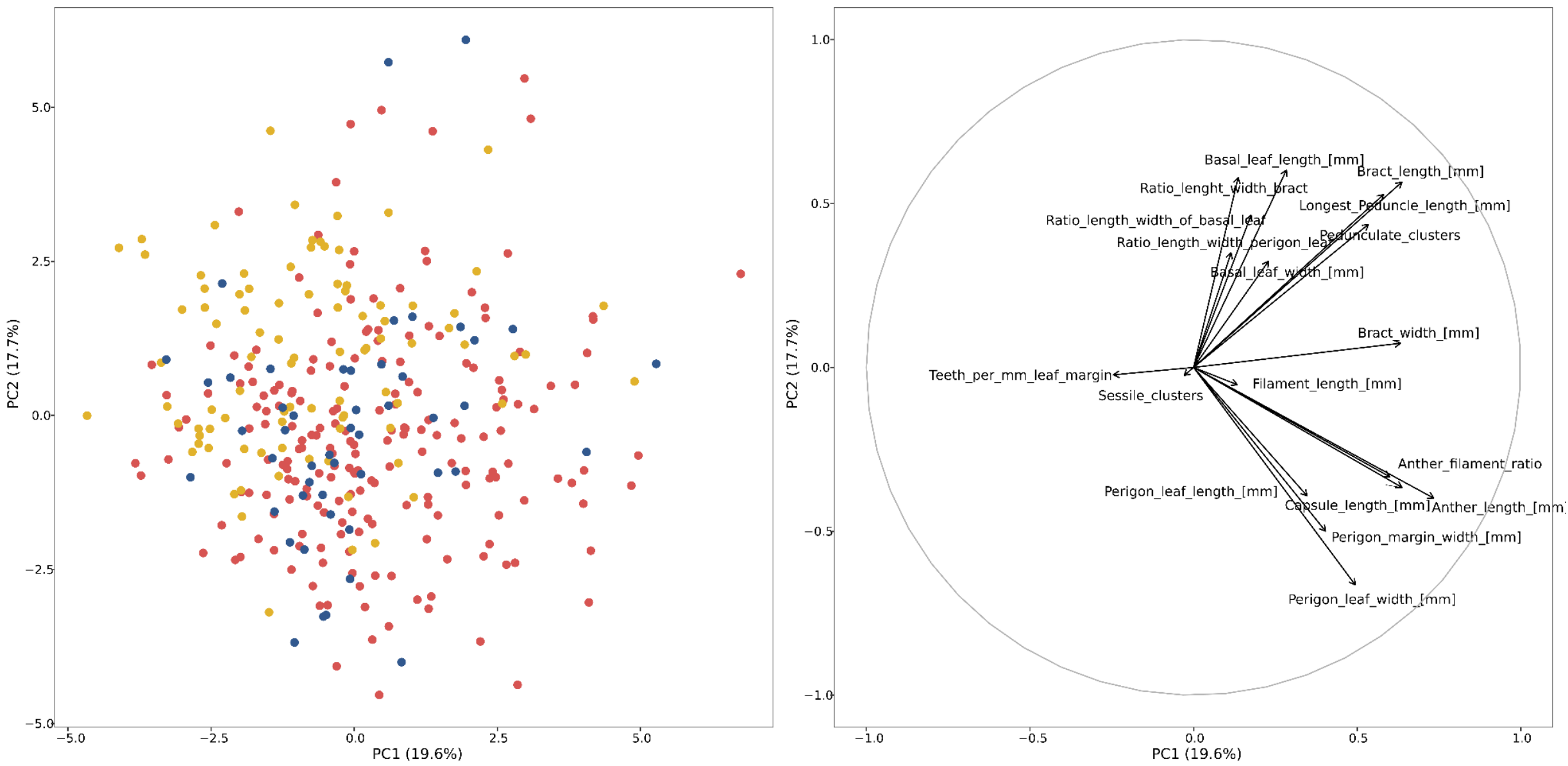

Fig. S20 Box plots of non-seed morphological characters of *Luzula exspectata* (EXS; yellow), *L. multiflora* (MUL; blue) and *L. alpina* (ALP; red). Thick horizontal lines are median values, boxes are interquartile ranges (IQR) and whiskers show 1.5 times the IQR. Asterisks above brackets indicate significant mean differences according to Kruskal-Wallis test followed by Dunn's *post hoc* test (ns: not significant, * $P < 0.05$, ** $P < 0.01$, *** $P < 0.001$).

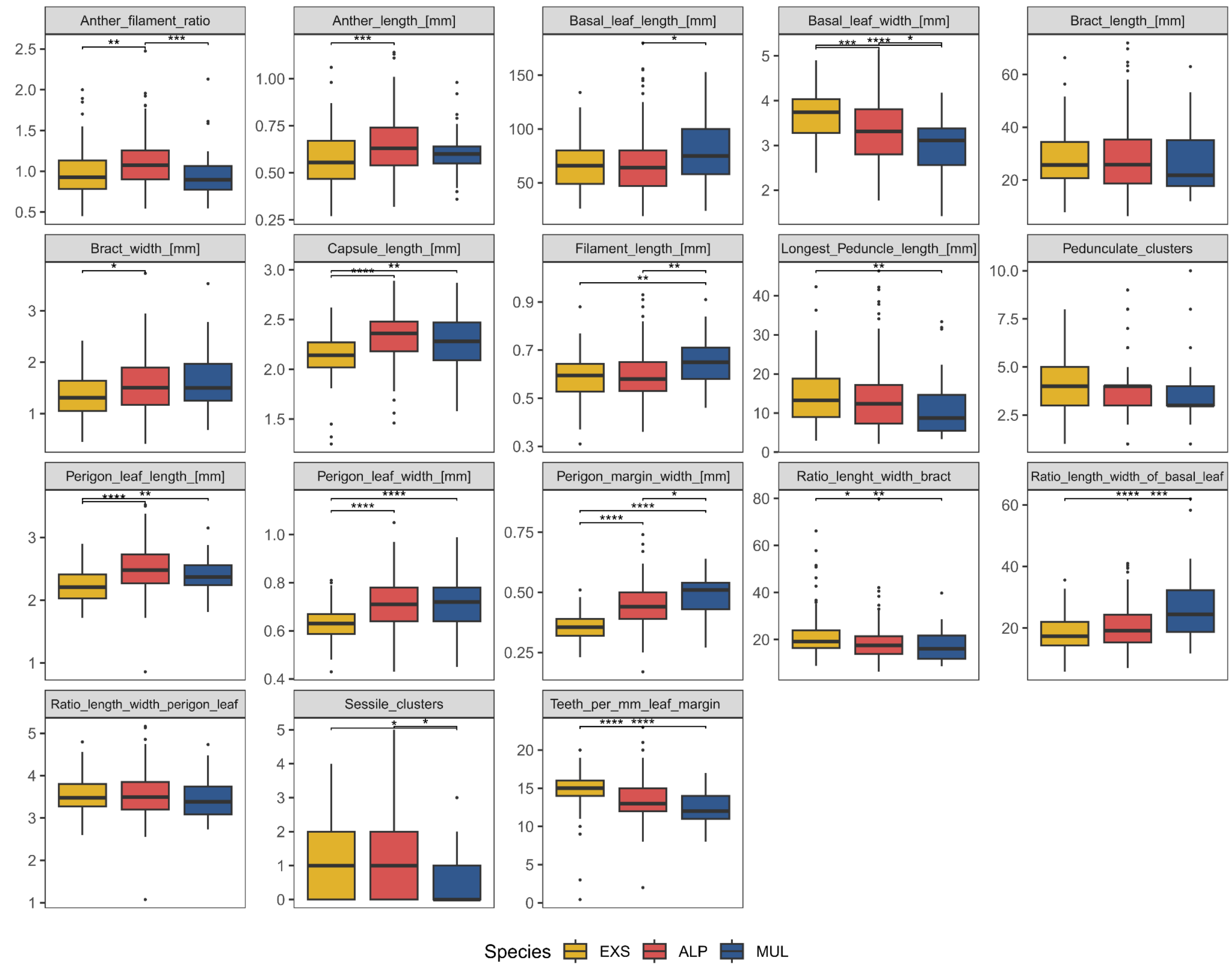

Fig. S21 Box plots of seed morphological characters of *Luzula exspectata* (EXS; yellow), *L. multiflora* (MUL; blue) and *L. alpina* (ALP; red). Thick horizontal lines are median values, boxes are interquartile ranges (IQR) and whiskers show 1.5 times the IQR. Asterisks above brackets indicate significant mean differences according to Kruskal-Wallis test followed by Dunn's *post hoc* test (ns: not significant, * $P < 0.05$, ** $P < 0.01$, *** $P < 0.001$).

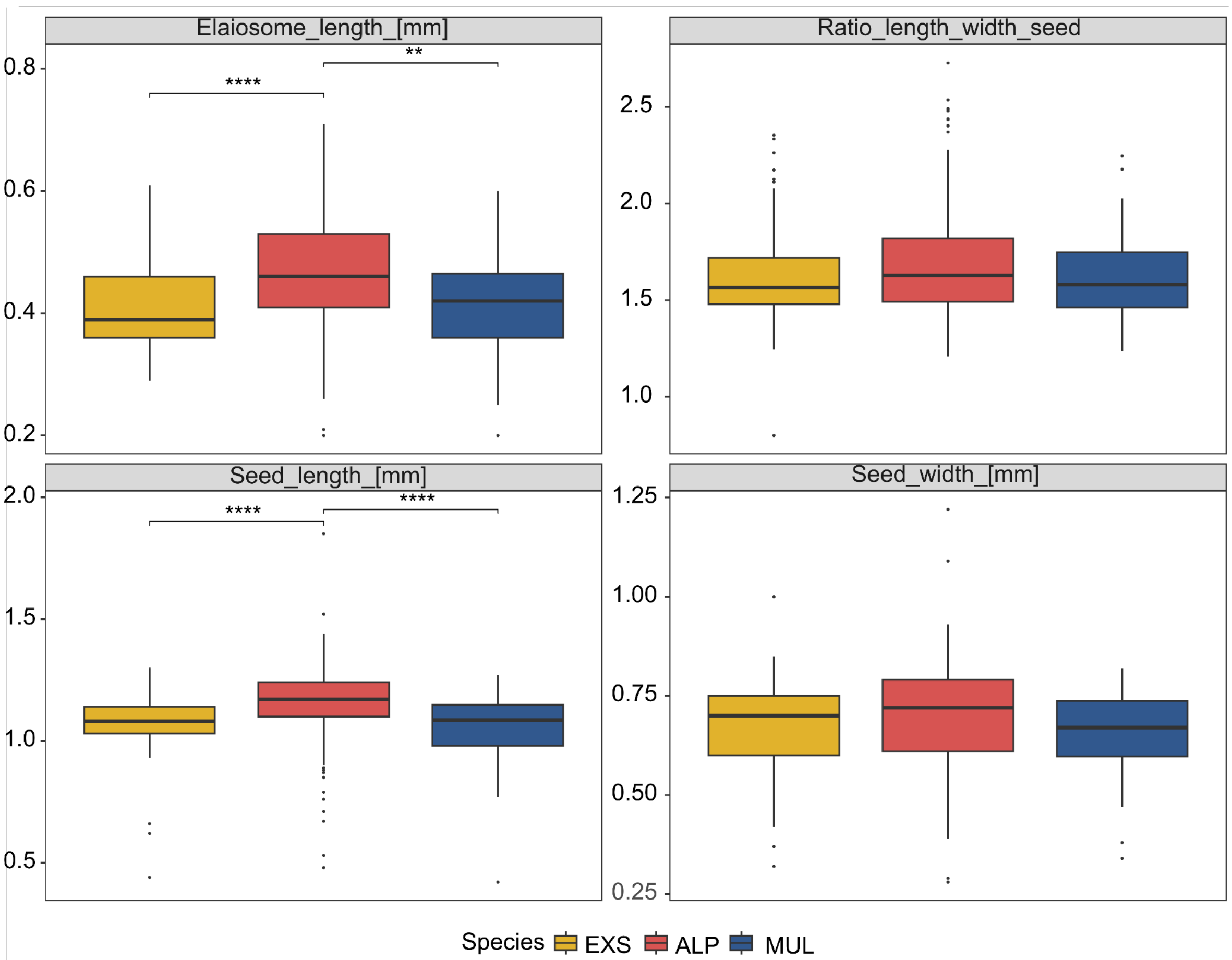

Fig. S22 Principal component analysis (PCA) of seed morphological characters of *Luzula exspectata* (EXS; yellow), *L. multiflora* (MUL; blue) and *L. alpina* (ALP; red).

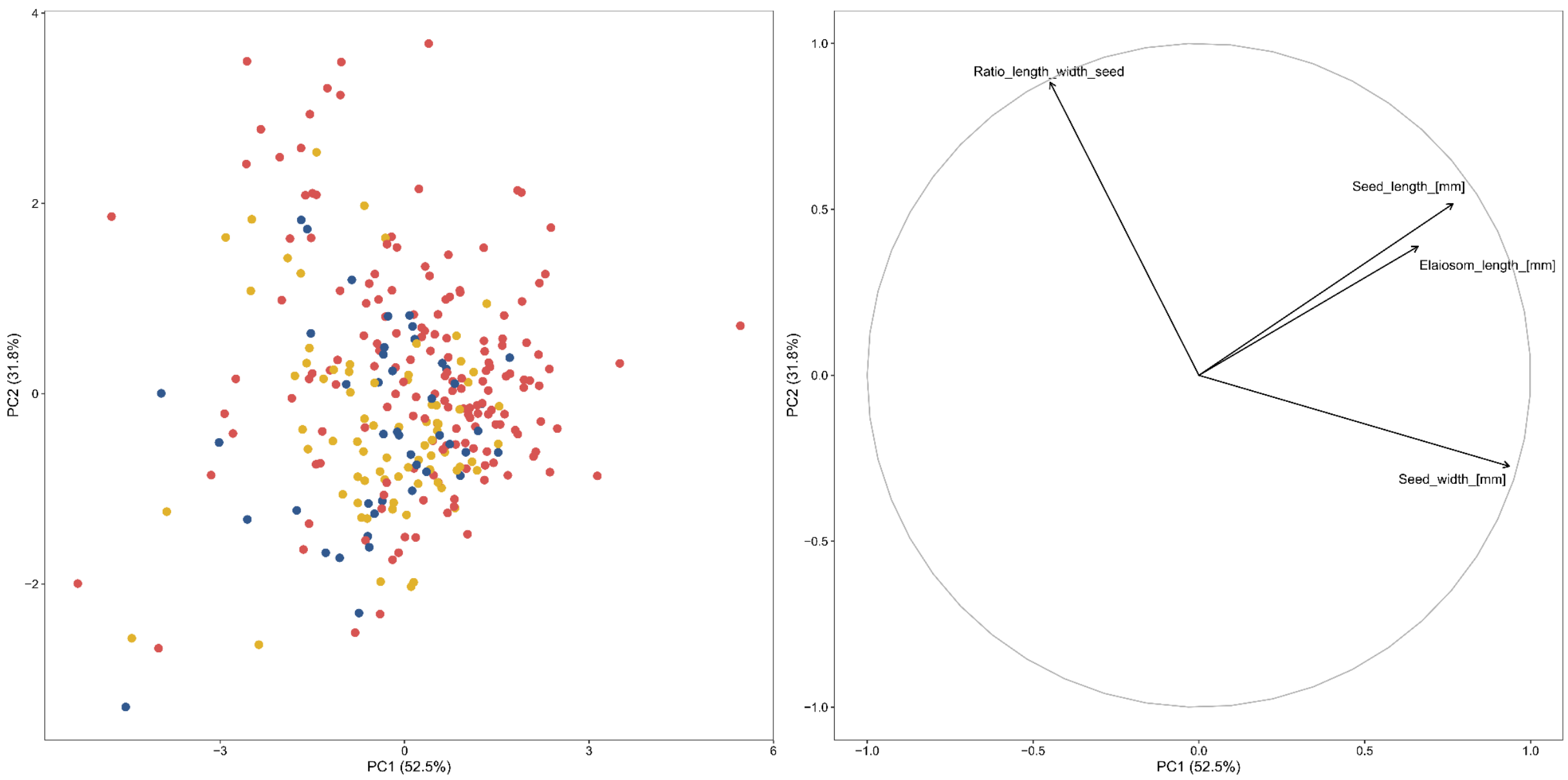

Fig. S23 Linear discriminant analysis (LDA) of seed morphological characters of *Luzula exspectata* (EXS; yellow), *L. multiflora* (MUL; blue) and *L. alpina* (ALP; red). Ellipses show 95%-quantiles of samples for each species.

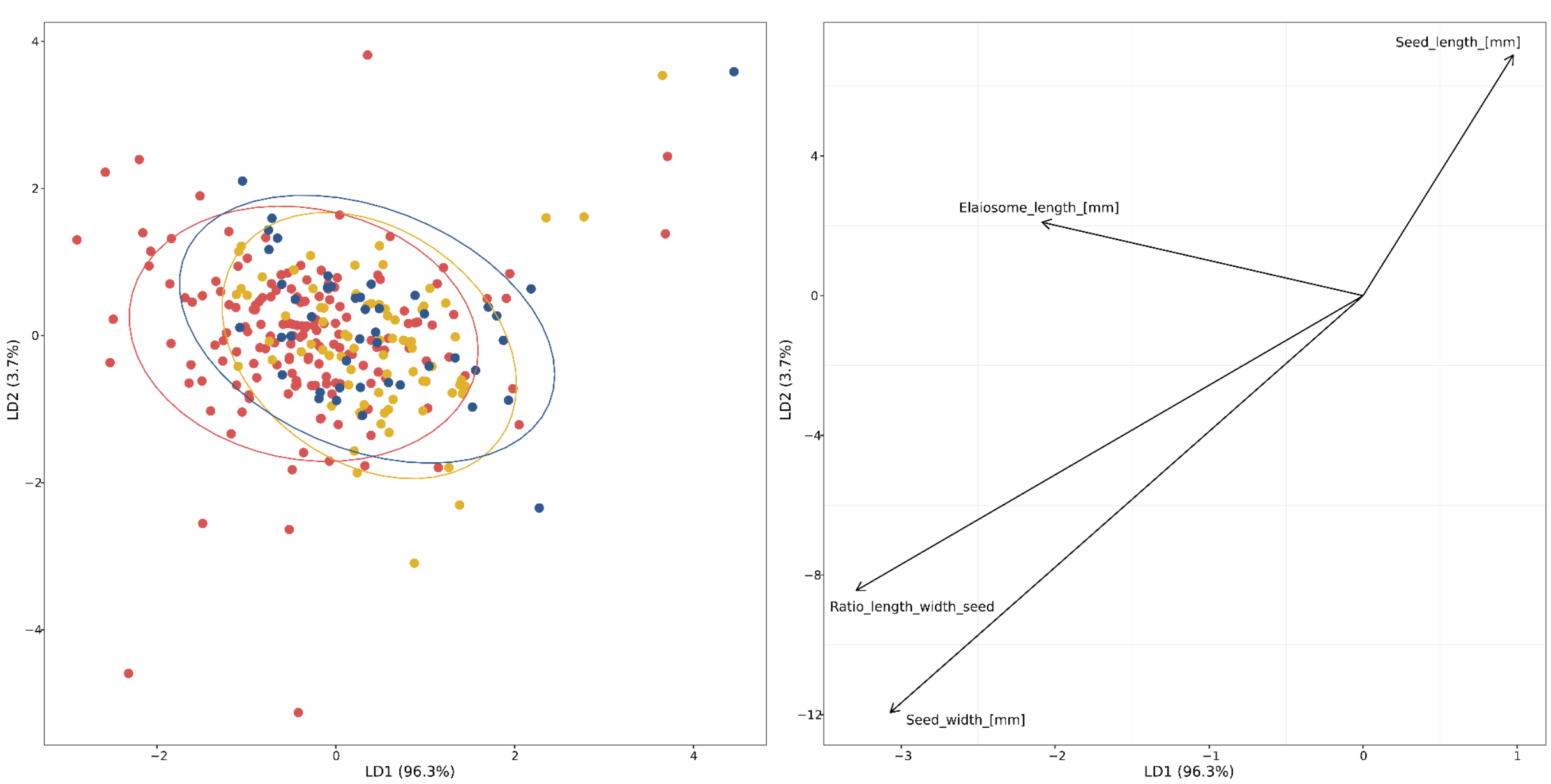

Table S1 *Luzula* accessions used in this study. This table is available as a separate spreadsheet

Table S2 Details of filtering applied to ddRADseq data and number of samples and single nucleotide polymorphims (SNPs) used for each analysis. ALP: *Luzula alpina*; EXS: *L. exspectata*; MUL: *L. multiflora*; QUAL: Phred-scaled quality score; QD: QualByDepth; MQ: RMSMappingQuality; MQRankSum: MappingQualityRankSumTest; ReadPosRankSum: ReadPosRankSumTest; minDP: minimum genotype read depth; maxDP: maximum genotype read depth; MAC: minor allele count.

Analysis	# of SNPs	# of samples	Filtering
Baseline VCF for all downstream analyses after quality filtering	840912	517	GATK hard filtering recommendations "QUAL < 40.0", "QD < 2.0", "MQ < 40.0", "MQRankSum < -12.5", "ReadPosRankSum < -15.0", Filter (and set to no call) genotypes with minDP < 8 and maxDP > 200
NeighbourNet & PCA – all species	49884	517	≤ 50% missing data
Hybrid index	2096	493	1 SNP per RAD locus, only SNPs that differed between EXS and MUL
STRUCTURE – balanced	1993	256	≤ 20% missing data, MAC ≥ 3, 1 SNP per RAD locus
STRUCTURE – full dataset	2070	517	≤ 20% missing data, MAC ≥ 3, 1 SNP per RAD locus
STRUCTURE – EXS	4288	121	≤ 20% missing data, MAC ≥ 3, 1 SNP per RAD locus
STRUCTURE – MUL	1815	83	≤ 20% missing data, MAC ≥ 3, 1 SNP per RAD locus
STRUCTURE – ALP	2302	306	≤ 20% missing data, MAC ≥ 3, 1 SNP per RAD locus
Range Expansion – EXS	2242	124	≤ 20% missing data, 1 SNP per RAD locus, including outgroup
Range Expansion – MUL	2242	86	≤ 20% missing data, 1 SNP per RAD locus, including outgroup
Range Expansion – ALP	2242	309	≤ 20% missing data, 1 SNP per RAD locus, including outgroup
Population statistics – EXS	5419	121	≤ 20% missing data, 1 SNP per RAD locus, min. 2 individuals per pop

Population statistics – MUL	1965	83	≤ 20% missing data, 1 SNP per RAD locus, min. 2 individuals per pop
Population statistics – ALP	2440	306	≤ 20% missing data, 1 SNP per RAD locus, min. 2 individuals per pop

Table S3 Climatic and edaphic variables used for environmental niche modelling (ENM) and niche analysis in *ecospat*.

ENM	Niche analysis	Abbreviation	Definition
1	1	bio1	Annual Mean Temperature [°C]
	1	bio2	Mean Diurnal Range [°C]
	1	bio3	Isothermality
	1	bio4	Temperature Seasonality [standard deviation]
	1	bio5	Max Temperature of Warmest Month [°C]
	1	bio6	Min Temperature of Coldest Month [°C]
	1	bio7	Temperature Annual Range [°C]
1	1	bio8	Mean Temperature of Wettest Quarter [°C]
	1	bio9	Mean Temperature of Driest Quarter [°C]
	1	bio10	Mean Temperature of Warmest Quarter [°C]
	1	bio11	Mean Temperature of Coldest Quarter [°C]
1	1	bio12	Annual Precipitation [mm/year]
	1	bio13	Precipitation of Wettest Month [mm/month]
	1	bio14	Precipitation of Driest Month [mm/month]
1	1	bio15	Precipitation Seasonality [coefficient of variation]
	1	bio16	Precipitation of Wettest Quarter [mm/quarter]
	1	bio17	Precipitation of Driest Quarter [mm/quarter]
	1	bio18	Precipitation of Warmest Quarter [mm/quarter]
	1	bio19	Precipitation of Coldest Quarter [mm/quarter]
1	1	bedrock_acidity	Bedrock acidity computed from Geo-Lim
	1	CEC	Cation exchange capacity at pH7 at 5-15cm soil depth [mmol(c)/kg]
	1	CLA	Clay content at 5-15cm soil depth [g/kg]
	1	CRF	Coarse fragments at 5-15cm soil depth [cm^3/dm^3]
	1	N	Nitrogen at 5-15cm soil depth [cg/kg]

1	pH	pH in water at 5-15cm soil depth [pH*10]
1	SAN	Sand content at 5-15 cm soil depth [g/kg]
1	SIL	Silt content at 5-15 cm soil depth [g/kg]
1	SOC	Soil organic carbon at 5-15 cm soil depth [dg/kg]

Table S4 Vegetation relevé data. This table is available as a separate spreadsheet.

Table S5 Fine-grained ecological data. This table is available as a separate spreadsheet.

Table S6 Taxa with no Karrer ecological indicator values (EIVs) available. Adapted EIVs were computed for four species by rescaling Landolt EIVs to the range of Karrer EIVs (8 records, 0.1% of data). Three species groups without EIV were excluded from niche analyses (170 records, 2.8% data). L: light availability; T: temperature; C: continentality; H: humidity; R: soil reaction; N: nitrogen availability.

**Taxon**	**adapted Landolt EIVs**							**no EIVs available**
	**L**	**T**	**C**	**H**	**R**	**N**	**# samples**	**# samples**
Hypericum richeri	5	5	4	5	9	5.4	1	
Gentianella ramosa	7	3	7	5	3.6	3.6	1	
Primula glaucescens	7	4	5	6	7.2	3.6	1	
Astrantia minor	5	5	5	5	3.6	3.6	5	
Alchemilla alpina agg.								19
Alchemilla vulgaris agg.								97
Hieracium pilosella agg.								54
Total							8 (0.1%)	170 (2.8%)

Table S7 Morphometric data. This table is available as a separate spreadsheet.

Table S8 Results of Mantel tests with 20,000 permutations to test for the effects of isolation by distance (IBD) and isolation by environment (IBE). IBD was inferred from correlations of genetic distances (rho/(rho-1)) and Euclidean geographic distances (G ~ Geo). IBE was calculated from the correlation of genetic distances and Euclidean distances from principal components of environmental space while accounting for spatial structure (G ~ E | Geo). Mantel r statistics and *P*-values are reported for all tests. *P*-values < 0.05 are indicated with bold font. ALP: *Luzula alpina*; EXS: *L. exspectata*; MUL: *L. multiflora*.

	**G ~ Geo (IBD)**		**G ~ E**		**G ~ E | Geo (IBE)**	
**Species**	**r**	***P*-value**	**r**	***P*-value**	**r**	***P*-value**
EXS	0.3904	**9.999e-05**	-0.08187	0.81932	-0.2245	0.9956
MUL	0.2403	0.09679	0.261	0.075092	0.1671	0.16218
ALP	0.4054	**9.999e-05**	0.4227	**9.999e-05**	0.3195	**9.999e-05**

Table S9 Results of multivariate redundancy analysis associating population-level allele frequencies with geographic coordinates and/or coordinates along the first two principal components of environmental space. Significance was assessed with 20,000 permutations. Isolation by distance (IBD) was inferred from correlations of allele frequencies and geographic coordinates (G ~ Geo). IBE was calculated from the correlation of allele frequencies and coordinates in environmental space while accounting for spatial structure (G ~ E | Geo). F statistics and *P*-values are reported for all tests. *P*-values < 0.05 are indicated with bold font. ALP: *Luzula alpina*; EXS: *L. exspectata*; MUL: *L. multiflora*.

	**G ~ Geo (IBD)**		**G ~ E**		**G ~ E | Geo (IBE)**	
**Species**	**F-statistic**	***P*-value**	**F-statistic**	***P*-value**	**F-statistic**	***P*-value**
EXS	2.748	**0.0000**	1.957	**0.0001**	1.255	**0.0435**
MUL	1.941	**0.0001**	1.456	**0.0176**	1.244	0.1340
ALP	4.703	**0.0000**	1.744	**0.0015**	1.719	**0.0002**

Table S10 Inferred approximate geographic origins and results of the test for geographic expansion for the entire species and the two major STRUCTURE clusters for *Luzula alpina* (ALP) and *L. exspectata* (EXS). *q* is the strength of the directionality index, R100 is the estimated decrease in genomic diversity over 100 km, and R^2 and *P* are the correlation coefficient and *P*-value for the most likely geographic origin, respectively. *P*-values < 0.05 are indicated with bold font.

Species	Region	Origin	q	R100	R^2	*P*
EXS	full	45.5758° N, 11.9111° E	0.0001	0.9806	0.0522	**1.24e-18**
	northeast	45.5000° N, 9.3444° E	0.0002	0.9633	0.1637	**1.86e-14**
	southwest	45.5000° N, 12.1444° E	0.0002	0.9474	0.1669	**7.43e-17**
MUL	full	46.8131° N, 13.8556° E	0.0004	0.9217	0.5617	**9.93e-36**
ALP	full	46.3838° N, 8.8000 ° E	0.0002	0.9615	0.1330	**3.49e-55**
	northwest	46.2071° N, 8.8000 ° E	0.0005	0.9162	0.4961	**6.67e-22**
	southeast	47.8737° N, 8.8000 ° E	0.0001	0.9764	0.0399	**1.98e-09**

Table S11 Summary of linear models to test for the relationship between population-average nucleotide diversity (π) and geographic distance to the inferred origin of range expansion. *P*-values < 0.05 are indicated with bold font. ALP: *Luzula alpina*; EXS: *L. exspectata*; MUL: *L. multiflora*; df: degrees of freedom.

Species	Model	Term	Estimate	Std. Error	t-value	*P*-value	df	adj. R^2	F-statistic
EXS	pi ~ distance	intercept	9.787e-02	7.075e-03	13.832	**< 2e-16**	43	0.3251	22.2
		distance	-1.853e-04	3.933e-05	-4.712	**2.59e-05**			
MUL	pi ~ distance	intercept	1.666e-01	5.079e-03	32.801	**2.22e-15**	15	0.631	28.35
		distance	-3.022e-07	5.675e-08	-5.325	**8.49e-05**			
ALP	pi ~ distance	intercept	1.068e-01	2.596e-03	41.155	**< 2e-16**	53	0.07181	5.177
		distance	-2.787e-05	1.225e-05	-2.275	**0.027**			

Table S12 Metrics of niche divergence computed from 32 climatic, topographic, lithologic and edaphic variables from the coarse-grained dataset for all pairwise comparisons among study species and between *Luzula alpina* (ALP) and the combined niche of *L. exspectata* (EXS) and *L. multiflora* (MUL). D: Schoener's *D*, I: Hellinger's *I*, P_{Deq}: *P*-value of the niche equivalency test for *D*, P_{Ieq}: *P*-value of the niche equivalency test for *I*, P_{Dsim}: *P*-value of the niche similarity test for *D*, P_{Isim}: *P*-value of the niche similarity test for *I*, B_{PC1}: niche breadth along PC1 (median ± standard deviation), B_{PC2}: niche breadth along PC2, S: niche stability, U: niche unfilling, E: niche expansion. Significant *P*-values < 0.05 are highlighted in bold. Niche breadth is given for the species indicated.

Comparison	D	I	P_{Deq}	P_{Ieq}	P_{Dsim}	P_{Isim}	B_{PC1}	B_{PC2}	S	U	E
MUL – EXS	0.54	0.73	**0.0110**	**0.0150**	**0.0500**	**0.0480**	EXS 2.07±0.13	EXS 2.54±0.12	0.54	0.00	0.46
EXS – ALP	0.75	0.91	0.2927	0.3816	**0.0460**	**0.0340**	ALP 1.83±0.11	ALP 2.04±0.14	0.99	0.13	0.01
MUL – ALP	0.63	0.79	0.0769	0.0949	**0.0280**	**0.0230**	MUL 1.37±0.07	MUL 1.47±0.07	0.66	0.00	0.34
EXS+MUL – ALP	0.78	0.93	0.3037	0.4565	**0.0410**	**0.0330**	1.95±0.13	2.34±0.13	0.99	0.10	0.00

Table S13 Analysis of variance (ANOVA) results for the optimised model, axes and explanatory variables of the redundancy analysis (RDA) of coarse-grained environmental variables. Df: degrees of freedom; N: Nitrogen at 5-15cm soil depth [cg/kg]; bio19: Precipitation of Coldest Quarter [mm/quarter]; pH: pH in water at 5-15cm soil depth [pH*10].

**RDA ANOVA model**	**Df**	**Variance**	**F value**	***P*-value**
Model	4	0.135	8.852	0.001
Residual	127	0.485		
**RDA ANOVA axes**	**Df**	**Variance**	**F value**	***P*-value**
RDA1	1	0.077	20.376	0.001
RDA2	1	0.059	15.590	0.002
Residual	129	0.485		
**RDA ANOVA explanatory variables**	**Df**	**Variance**	**F value**	***P*-value**
N	1	0.046	12.115	0.001
bedrock_acidity	1	0.040	10.453	0.001
bio19	1	0.025	6.519	0.010
pH	1	0.024	6.320	0.003
Residual	127	0.485		

Table S14 Analysis of variance (ANOVA) results for the optimised model, axes and explanatory variables of the redundancy analysis (RDA) of accompanying plant species.

**RDA ANOVA model**	**Df**	**Variance**	**F value**	***P*-value**
Model	49	0.231	6.655	0.001
Residual	455	0.323		
**RDA ANOVA axes**	**Df**	**Variance**	**F value**	***P*-value**
RDA1	1	0.151	234.23	0.001
RDA2	1	0.081	125.56	0.001
Residual	502	0.323		
**RDA ANOVA explanatory variables**	**Df**	**Variance**	**F value**	***P*-value**

Nardus stricta	1	0.013	19.012	0.001
Kalmia procumbens	1	0.010	14.198	0.001
Carlina acaulis	1	0.010	13.739	0.001
Parnassia palustris	1	0.008	11.906	0.001
Astrantia bavarica	1	0.008	11.231	0.001
Euphrasia officinalis	1	0.008	10.690	0.001
Carex atrata	1	0.008	10.997	0.001
Festuca nitida	1	0.007	10.506	0.001
Vaccinium myrtillus	1	0.007	9.807	0.002
Soldanella pusilla	1	0.007	9.944	0.002
Helictochloa versicolor	1	0.007	10.354	0.001
Achillea millefolium agg.	1	0.006	9.054	0.001
Potentilla crantzii	1	0.006	7.934	0.001
Lathyrus pratensis	1	0.006	8.856	0.001
Carex flacca subsp. *flacca*	1	0.006	7.984	0.003
Pulsatilla alpina subsp. *alba*	1	0.006	7.786	0.001
Rumex acetosella	1	0.003	4.443	0.001
Lupinus polyphyllus	1	0.006	8.152	0.001
Ranunculus carinthiacus	1	0.005	7.045	0.001
Geum montanum	1	0.005	7.525	0.001
Hieracium pilosella agg.	1	0.005	7.401	0.003
Cirsium spinosissimum	1	0.004	5.275	0.001
Pilosella aurantiaca	1	0.003	4.916	0.001
Dianthus alpinus	1	0.003	4.903	0.006
Koeleria macrantha	1	0.004	5.733	0.001
Calluna vulgaris	1	0.004	5.184	0.011
Carex montana	1	0.003	4.807	0.014
Potentilla erecta	1	0.003	4.698	0.005
Avenula pubescens	1	0.003	3.891	0.026
Heliosperma alpestre	1	0.003	4.177	0.012
Trifolium pallescens	1	0.003	4.560	0.016
Cerastium arvense	1	0.003	4.825	0.012
Carex ornithopoda	1	0.002	3.362	0.046
Hieracium pilosum	1	0.003	4.186	0.016

Vaccinium vitis-idaea	1	0.003	4.339	0.017
Leucanthemum adustum	1	0.003	4.794	0.007
Cerastium fontanum	1	0.003	4.312	0.018
Potentilla brauneana	1	0.004	4.945	0.001
Cerastium carinthiacum	1	0.003	3.763	0.030
Betonica alopecuros	1	0.003	3.814	0.016
Melampyrum sylvaticum	1	0.002	3.363	0.036
Primula auricula	1	0.003	4.061	0.010
Koeleria pyramidata	1	0.002	3.115	0.037
Veronica beccabunga	1	0.003	3.531	0.001
Omalotheca sylvaticum	1	0.002	3.307	0.001
Sagina saginoides	1	0.002	3.337	0.038
Pimpinella saxifraga	1	0.003	3.600	0.001
Cerastium holosteoides	1	0.003	3.546	0.032
Thymus praecox subsp. *praecox*	1	0.002	3.204	0.036
Residual	455	0.323		

Table S15 Metrics of niche divergence computed from 14 environmental predictors of the fine-grained dataset, including community-weighted means of six Karrer indicator values, for all pairwise comparisons among study species and between *Luzula alpina* (ALP) and the combined niche of *L. exspectata* (EXS) and *L. multiflora* (MUL). D: Schoener's *D*, I: Hellinger's *I*, P_{Deq}: *P*-value of the niche equivalency test for *D*, P_{Ieq}: *P*-value of the niche equivalency test for *I*, P_{Dsim}: *P*-value of the niche similarity test for *D*, P_{Isim}: *P*-value of the niche similarity test for *I*, B_{PC1}: niche breadth along PC1 (median ± standard deviation), B_{PC2}: niche breadth along PC2, S: niche stability, U: niche unfilling, E: niche expansion. Significant *P*-values < 0.05 are highlighted in bold. Niche breadth is given for the species indicated.

**Comparison**	**D**	**I**	P_{Deq}	P_{Ieq}	P_{Dsim}	P_{Isim}	B_{PC1}	B_{PC2}	**S**	**U**	**E**
MUL – EXS	0.71	0.88	**0.0020**	**0.0010**	0.3157	0.3377	EXS<br>1.53±0.08	EXS<br>1.23±0.10	0.97	0.15	0.03
EXS – ALP	0.67	0.89	**0.0020**	**0.0020**	0.1728	0.1728	ALP<br>1.69±0.11	ALP<br>1.24±0.11	0.91	0.00	0.09
MUL – ALP	0.75	0.93	**0.0460**	**0.0180**	0.1698	0.2368	MUL<br>1.80±0.10	MUL<br>1.40±0.10	0.96	0.03	0.04
EXS+MUL – ALP	0.74	0.94	**0.0010**	**0.0060**	0.0969	0.1279	1.74±0.10	1.38±0.11	0.98	0.02	0.02

Table S16 Analysis of variance (ANOVA) results for the optimised model, axes and explanatory variables of the redundancy analysis (RDA) of fine-grained environmental variables. Df: degrees of freedom; Reaction (R): community-weighted mean Karrer indicator value for soil reaction.

**RDA ANOVA model**	**Df**	**Variance**	**F value**	***P*-value**
Model	5	0.069	14.21	0.001
Residual	499	0.485		
**RDA ANOVA axes**	**Df**	**Variance**	**F value**	***P*-value**
RDA1	1	0.052	53.969	0.001
RDA2	1	0.017	17.504	0.001
Residual	502	0.485		
**RDA ANOVA explanatory variables**	**Df**	**Variance**	**F value**	***P*-value**
Reaction (R)	1	0.035	35.944	0.001
Elevation	1	0.017	17.969	0.001
CryptogamCover	1	0.009	9.173	0.001
OrganicCover	1	0.004	4.401	0.011
Northness	1	0.003	3.559	0.036
Residual	499	0.486		

Methods S1 Isolation-by-distance and isolation-by-environment

We explored the impact of demographic and ecological processes on the spatial structure of genetic variation by computing isolation-by-distance (IBD) and isolation-by-environment (IBE) for each species (Wang *et al.*, 2013). Following Huynh *et al.* (2020), we inferred patterns of IBD by comparing Euclidean geographic and genetic distances (rho/(rho-1) with Mantel tests using 20,000 permutations in *vegan*. Signatures of IBE are expected if gene flow occurs predominantly among individuals from similar ecological conditions (Wang & Bradburd, 2014) and were tested through partial Mantel tests of genetic distances and Euclidean distances from principal components (PCs) of environmental space derived from the coarse-grained dataset while accounting for spatial structure. We validated patterns of IBD and IBE through partial RDA with 20,000 permutations, associating population-level allele frequencies with coordinates along the first two PCs of environmental space while accounting for geographical isolation.

Methods S2 Environmental data acquisition

We combined climatic, topographic, lithologic and edaphic data to assess differences in coarse-grained abiotic ecological niches among our three study species. Climatic variables were retrieved at 30-arc second resolution from CHELSA (Karger *et al.*, 2017). A digital elevation model (DEM) for Europe at 30 m resolution was obtained from the GMES RDA project (EU-DEM; http://data.europa.eu/88u/dataset/f576cda8-d598-478c-b8fe-ad2634c927e8) and used to compute the topographic variables elevation, slope, northness and topographic roughness, which were aggregated to 1 km resolution. Lithological data from Geo-LiM (Donnini *et al.*, 2020) were used to construct a bedrock-type layer following Chauvier *et al.* (2021). First, soil lithology was classified into three categories: calcareous, siliceous, and mixed (Table Bedrock Classes). In a second step, this newly created layer was converted to a 100 m grid for each category. Finally, all 100 m grids were aggregated to 1 km resolution by averaging the proportions of the three categories within each grid cell. Topsoil data that are ecologically relevant for plant species were retrieved from SoilGrids (Hengl *et al.*, 2017). Climatic data for the Last Glacial Maximum used to project ENMs to paleoclimatic conditions were retrieved from CHELSA for four general circulation models (IPSL-CM5A-LR, CCSM4, MRI-CGCM3, MPI-ESM-P).

Bedrock Classes: Conversion of Geo-LIM lithological categories into bedrock classes and corresponding bedrock acidity (ranging from 0 to 1).

Geo-LIM category	Bedrock class	Bedrock acidity
acid rocks	siliceous	1
claystone	siliceous	1
mafic rocks	mixed	0.5
sandstone	siliceous	1
intermediate rock	mixed	0.5
gypsum evaporite	calcareous	0
mix carbonate rocks	calcareous	0
pure carbonate rocks	calcareous	0
metamorphic rocks	mixed	0.5

Notes S1 Analyses of vegetation data

The communities of accompanying plant species were strongly overlapping among our study species, as reflected by the lack of separation in the DCA and TWINSPAN clusters that did not align with the three *Luzula* species (Fig. 4a). Characteristic species for the two major TWINSPAN clusters were *Nardus stricta* on the one hand, and *Bistorta vivipara*, *Carex sempervirens*, *Galium anisophyllon*, *Lotus corniculatus*, *Sesleria caerulea* and *Trifolium pratense* on the other hand. The first and larger (n = 356) TWINSPAN cluster consisted mainly of ALP (65%), whereas MUL (19%) and EXS were scarcer (15%). Even though ALP was still the dominant species (49%) of the second and smaller (n = 149) cluster, EXS reached a comparable prevalence (42%), while MUL remained rare (9%). Most individuals of ALP (76%) and MUL (83%) belonged to the first TWINSPAN cluster, whereas accessions of EXS were more evenly distributed between the two clusters (47% and 53%, respectively.)

Although the accompanying plant communities were overall similar among the three study species, redundancy analysis (RDA) revealed a number of diagnostic species, which in case of EXS

were mostly characteristic for alpine grasslands over limestone (e.g., *Betonica alopecurus*, *Koeleria pyramidata*, *Potentilla crantzii*). In contrast, occurrences of ALP were characterised by the presence of species typical for acidic alpine meadows (e.g., *Helictochloa versicolor*, *Nardus stricta*, *Potentilla erecta*), and MUL co-occurred with acidophilic species of alpine dwarf-shrub heaths (e.g., *Kalmia procumbens*, *Vaccinium myrtillus*, *Vaccinium vitis-idaea*). Together, the environmental preferences of our study species inferred from both coarse- and fine-grained analyses were consistent with previous findings (Bačič *et al.*, 2019; Geurden *et al.*, 2025) and characterised EXS as a species of alpine grasslands on calcareous substrates in areas with high snowfall. In contrast, MUL thrives in dwarf-shrub heaths with more acidic soil and better nitrogen availability. Lastly, ALP occurs predominantly in alpine meadows at high elevations over siliceous bedrock.

Notes S2 Idiosyncratic patterns of refugial areas and range expansion

Combining evidence from species distribution modelling, spatial patterns of ψ and private alleles, we inferred mostly peripheral refugia over limestone for EXS (Figs. 2, 3), which is in line with its preference for calcareous bedrock. These refugia were distributed partly along the southeastern margin of the Alps, a region that acted as an important Pleistocene refugium for many plant species (Tribsch & Schönswetter, 2003; Schönswetter *et al.*, 2005). In contrast, suitable habitat during the LGM coincided with known refugia for silicicolous plants in the Eastern Alps in case of MUL, but was more widespread along the periphery of the Alps for ALP, and spatial patterns of allele frequencies supported these results. Recolonisation of the Alps from glacial refugia followed idiosyncratic patterns for the three species as inferred from spatial patterns of ψ and π (Fig. 2). EXS expanded from the southern margin of the Alps, whereas the spread of ALP likely started somewhere along the western margin of the Eastern Alps, suggesting that the species may have persisted also within refugia in the Western Alps. Finally, MUL did not expand far beyond its refugial areas. All three species exhibited low genetic differentiation among populations which is likely explained by wind-pollination enabling gene flow across large distances (Kuzmanović *et al.*, 2017).

References

Bačič T, Dolenc Koce J, Frajman B. **2019**. Diversification and distribution patterns of *Luzula* sect. *Luzula* (Juncaceae) in the Eastern Alps: a cytogenetic approach combined with extensive herbarium revisions. *Alpine Botany* **129**: 149–161.

Chauvier Y, Thuiller W, Brun P, Lavergne S, Descombes P, Karger DN, Renaud J, Zimmermann NE. **2021**. Influence of climate, soil, and land cover on plant species distribution in the European Alps. *Ecological Monographs* **91**: e01433.

Donnini M, Marchesini I, Zucchini A. 2020. Geo-LiM: a new geo-lithological map for Central Europe (Germany, France, Switzerland, Austria, Slovenia, and Northern Italy) as a tool for the estimation of atmospheric CO2 consumption. *Journal of Maps* **16**: 43–55.

Geurden J, Heimer V, Frajman B. **2025**. Co-occurring *Luzula* species (Juncaceae) of different ploidies exhibit weak ecological differentiation at local scale in alpine grasslands of the Eastern Alps. *Alpine Botany* **135**: 275–288.

Hengl T, Mendes De Jesus J, Heuvelink GBM, Ruiperez Gonzalez M, Kilibarda M, Blagotić A, Shangguan W, Wright MN, Geng X, Bauer-Marschallinger B, et al. 2017. SoilGrids250m: Global gridded soil information based on machine learning. *PLOS ONE* **12**: e0169748.

Huynh S, Broennimann O, Guisan A, Felber F, Parisod C. **2020**. Eco-genetic additivity of diploids in allopolyploid wild wheats. *Ecology Letters* **23**: 663–673.

Karger DN, Conrad O, Böhner J, Kawohl T, Kreft H, Soria-Auza RW, Zimmermann NE, Linder HP, Kessler M. 2017. Climatologies at high resolution for the earth's land surface areas. *Scientific Data* **4**, 1–20.

Kuzmanović N, Lakušić D, Frajman B, Alegro A, Schönswetter P. **2017**. Phylogenetic relationships in Seslerieae (Poaceae) including resurrection of *Psilathera* and *Sesleriella*, two monotypic genera endemic to the Alps. *TAXON* **66**: 1349–1370.

Schönswetter P, Stehlik I, Holderegger R, Tribsch A. **2005**. Molecular evidence for glacial refugia of mountain plants in the European Alps. *Molecular Ecology* **14**: 3547–3555.

Tribsch A, Schönswetter P. **2003**. Patterns of endemism and comparative phylogeography confirm palaeo-environmental evidence for Pleistocene refugia in the Eastern Alps. *TAXON* **52**: 477–497.

Wang IJ, Bradburd GS. **2014**. Isolation by environment. *Molecular Ecology* **23**: 5649–5662.

Wang IJ, Glor RE, Losos JB. **2013**. Quantifying the roles of ecology and geography in spatial genetic divergence (F Adler, Ed.). *Ecology Letters* **16**: 175–182.
